# Inosine promotes erythrocyte metabolic reprogramming and restores oxygen release for rejuvenation via 2,3-BPG-PNP axis

**DOI:** 10.1038/s41421-026-00877-6

**Published:** 2026-03-17

**Authors:** Wuping Liu, Zhaoyu Yang, Changhan Chen, Fang Yu, Mengzhi Wu, Zhouzhou Yao, Yuhua Fan, Tingting Xie, Linlin Wan, Tiansheng Chou, Xianjing Feng, Hao Qi, Yuyu Chou, Juan Zhao, Juan Liu, Zhiyu Yang, Yujin Zhang, Rodney E. Kellems, Yang Xia

**Affiliations:** 1 National Medical Metabolomics International Collaborative Research Center, Xiangya Hospital, Central South University, Changsha, Hunan China; 2 National Clinical Research Center for Geriatric Disorders, Xiangya Hospital, Central South University, Changsha, Hunan China; 3 Institute of Integrative Medicine, Department of Integrated Traditional Chinese and Western Medicine, Xiangya Hospital, Central South University, Changsha, Hunan China; 4 Department of Neurology, Xiangya Hospital, Central South University, Changsha, Hunan China; 5 Department of Otolaryngology Head and Neck Surgery, Xiangya Hospital, Central South University, Changsha, Hunan China; 6 Department of General Medicine, Xiangya Hospital, Central South University, Changsha, Hunan China; 7 Department of Radiology; Xiangya Hospital, Central South University, Changsha, Hunan China; 8 Department of Laboratory Medicine, Xiangya Hospital, Central South University, Changsha, Hunan China; 9 Department of Biochemistry and Molecular Biology, The University of Texas McGovern Medical School at Houston, Houston, TX USA; 10 Furong Laboratory, Changsha, Hunan, China

**Keywords:** Mechanisms of disease, Ageing, Reprogramming

## Abstract

Aging-related diseases are aggravated by tissue hypoxia; however, the underlying mechanism remains unknown. Here, we report that the oxygen (O_2_) release capacity of red blood cells (RBCs) gradually decreases with age and is closely associated with aging-related tissue dysfunction. Metabolomic profiling of human and mouse RBCs and genetic studies in mice revealed that the reduction in 2,3-bisphosphoglyceric acid (2,3-BPG) content mediated by a decrease in bisphosphoglycerate mutase (BPGM) activity is a metabolic checkpoint underlying decreased RBC O_2_ release capability and dysfunction with advancing age. When glucose metabolism is impaired, erythroid inosine, transported by equilibrative nucleoside transporter 1 and converted to ribose 1-phosphate by increased purine nucleoside phosphorylase (PNP) activity, is an important compensatory fuel for RBCs during aging. In a preclinical study, inosine supplementation successfully alleviated the age-dependent reduction in BPGM activity that mediates glucose metabolic impairment, decreased O_2_ delivery, and tissue dysfunction. Finally, we unexpectedly discovered that 2,3-BPG acts as an inhibitor of PNP in RBCs by competing with the phosphate (Pi)-binding domain and interacting with residues serine 33 and alanine 116. Our studies revealed that impaired glucose metabolic reprogramming resulting from decreased BPGM activity underlies RBC bioenergetic decline and is a novel hallmark of aging. As 2,3-BPG levels decrease during aging, its inhibitory effect on PNP is reduced, resulting in increased PNP activity and inosine catabolism as an alternative fuel, suggesting that inosine is a potential rejuvenating therapy.

## Introduction

The aging population is increasing rapidly, overwhelming health care systems and creating an economic burden worldwide^1^. Elderly individuals are widely considered to have increased susceptibility to multiple diseases, including cardiovascular disease, obesity, metabolic disorders, and neurodegenerative diseases^2–4^. Given that aging-related diseases are frequently driven by tissue microenvironment hypoxia^5^, extensive research has been devoted to understanding the cellular and molecular mechanisms underlying tissue response to hypoxia during aging^6–8^. Hypoxia-induced transcription factors (HIFs) orchestrate changes in the expression of genes involved in vascular angiogenesis, metabolism, and immune function to combat hypoxia-mediated tissue damage and the progression of aging^9–12^. However, the specific cellular and molecular mechanisms initiating and driving age-dependent tissue hypoxia remain unknown. Additionally, how to mitigate age-related tissue microenvironment hypoxia, slow the onset of functional decline, and promote healthy aging are extremely challenging in both basic and clinical research.

Mature red blood cells (RBCs), the most abundant and only cell type that delivers oxygen (O_2_) in the body, are extremely sensitive to hypoxia and can release more O_2_ in response to both physiological hypoxia and pathological hypoxia^13–15^. Recent studies have made significant progress in revealing that although they lack a nucleus, mitochondria, and other complex organelles, RBCs are well equipped to promote O_2_ release in normal individuals under high-altitude hypoxia or in patients with pathological hypoxia owing to tightly regulated HIF-independent but glucose-dependent metabolic reprogramming^16–19^. However, owing to a lack of transcriptional activity, specific age-induced changes in RBCs cannot be effectively detected by current high-throughput omics profiling methods, such as transcriptomics and single-cell sequencing^20–22^. Although research regarding RBC metabolomics in the context of aging and related diseases is limited, emerging studies highlight the metabolic changes that occur in RBCs during aging. For example, using high-throughput metabolomics, D’Alessandro’s team revealed that glycolysis is influenced by sex, age, and race in stored RBCs and confirmed that ATP and hypoxanthine can serve as biomarkers for the recovery of RBC function post-transfusion^18^. Independent metabolomics research revealed that arginine metabolism in RBCs is a biomarker of organismal aging^23^, suggesting new potential targets for addressing the sequelae of aging. Notably, early parabiosis studies have indicated that multiple plasma circulating molecules, including chemokines, proteins, and enzymes, present in young blood have the potential for rejuvenation^24^. However, functional and metabolic changes that occur in erythrocytes, including their ability to deliver O_2_ and act as a hypoxia sensor, during natural aging, the specific mechanisms underlying these functional and metabolic alterations in RBCs in response to aging and their contribution to aging-related tissue hypoxia and dysfunction remain unclear. Thus, a specific means to target RBC function and counteract aging-related tissue functional decline is lacking.

Therefore, we sought to fill these important knowledge gaps with the goal of promoting healthy aging by employing multidisciplinary approaches, including accurately defining RBC O_2_ delivery capability in a large human cohort across the lifespan, multiple sophisticated genetically modified mice, nonbiased high-throughput metabolomic profiling, cutting-edge isotopic labeling and tracing experiments with inosine, preclinical studies, molecular modeling, mutagenesis and competitive enzymology assays. Here, we define decreased erythrocyte bioenergetics as a new hallmark of aging; reveal new significant systemic, cellular, metabolic, and molecular insights into age-induced RBC metabolic and functional decline and their contribution to aging-induced peripheral tissue hypoxia and dysfunction; and develop an innovative approach to increase RBC metabolism and O_2_ delivery for rejuvenation. Overall, our study highlights a new but compelling concept that “aging is not merely peripheral tissue hypoxia and dysfunction but also a systemic erythroid hypoxia–metabolic syndrome” and that targeting RBCs is a new approach to promote healthy aging.

## Results

### Decreased RBC oxygen delivery capacity is an early hallmark of aging linked to age-dependent tissue dysfunction

**Fig. 1:**
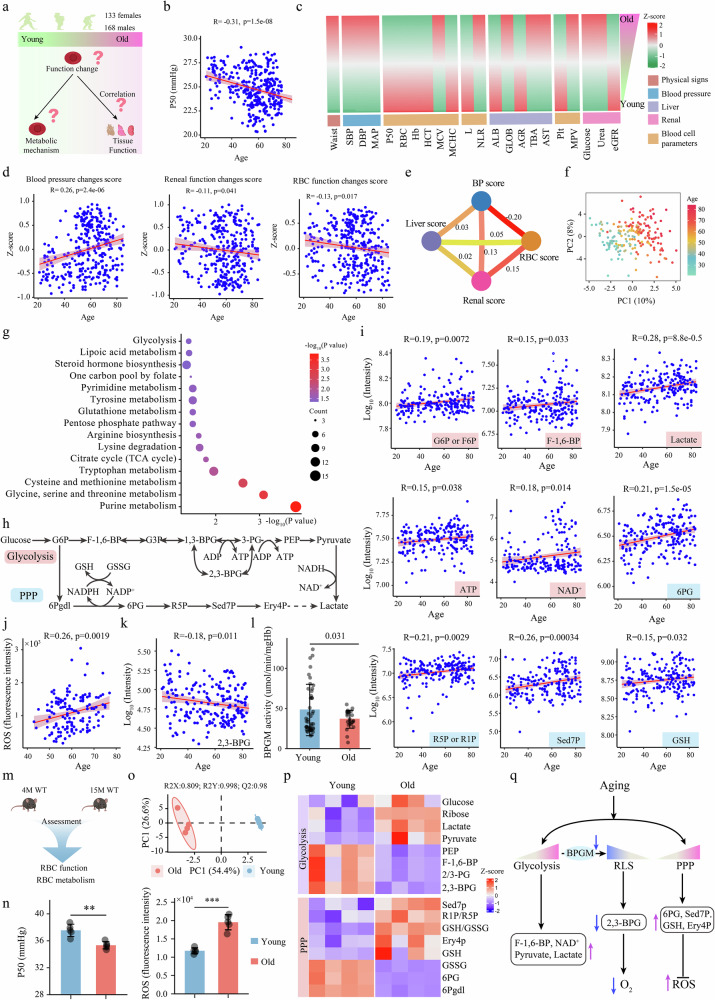
Age-dependent changes in RBC function and metabolism are linked to changes in tissue function. **a** Design of the human cohort study of RBC function and clinical changes during aging. **b** Changes in oxygen release capacity (P50) during aging. R represents the Pearson correlation coefficient. *P* represents the *P*-value for the Pearson correlation coefficient, and *P* < 0.05 indicates that the variable is significantly correlated with age. **c** Heatmap of the trajectories of different clinical parameters with age. The trajectory heatmap of aging-related clinical parameters was constructed by linear regression analysis. **d** Correlation analysis of clinical function scores and age. Clinical function scores were used to evaluate functional changes during aging. The lower the score is, the worse the functionality, and the higher the score is, the better the functionality. The clinical function scores were computed by the GSVA R package on the basis of the aging-related clinical function parameters. The clinical function parameters are shown in Supplementary Fig. S1. **e** Relationships between clinical function scores. The numbers represent the Pearson correlation. The specific details are shown in Supplementary Table S2. **f** PLS-DA score plot of RBC metabolic fingerprints during aging. **g** Pathways enriched in aging-related metabolites. **h**, **i** Changes in glycolysis and PPP intermediates in erythrocytes were revealed by untargeted metabolomics. **j** Changes in RBC ROS levels with age in a newly recruited group of 143 healthy individuals. **k** Changes in 2,3-BPG levels during aging. **l** Differences in BPGM enzyme activity in RBCs between the young (20–45 years) and elderly (60–85 years) groups. **m** Evaluation of RBC function and key metabolites in aged mice. This experiment included 4-month-old mice (young) and 15-month-old (old) WT mice. **n** Changes in the P50 and ROS levels between young and aged mice. *n* = 5. Data are presented as mean ± SD. **P < 0.01, ***P < 0.001, ^###^*P* < 0.001. **o** PLS-DA score plot of RBC metabolic fingerprints between young and aged mice. **p** Changes in erythrocyte glycolysis and PPP pathway intermediate metabolites between young and aged mice. **q** Summary of RBC glycolysis, RLS, and PPP metabolic reprogramming during aging. With aging, glycolysis and PPP activity increase, whereas RLS activity decreases. SBP systolic blood pressure, DBP diastolic blood pressure, MAP mean arterial pressure, HCT hematocrit, Hb hemoglobin, MCV mean corpuscular volume, MCH mean corpuscular hemoglobin, MCHC mean corpuscular hemoglobin concentration, RDW red blood cell distribution width, WBC white blood cell, Plt platelet, N neutrophil, L lymphocyte, Eso eosinophil, Baso basophil, Mono monocyte, PCT procalcitonin, MPV mean platelet volume, PDW platelet distribution width, TP total protein, ALB albumin, GLOB globulin, AG ratio albumin‒globulin ratio, TBIL total bilirubin, DBIL direct bilirubin, TBA total biliary acid, ALT alanine transaminase, AST aspartate aminotransferase, eGFR estimated glomerular filtration rate, UA uric acid, HbA1C glycated hemoglobin, TG triglyceride, TC total cholesterol, HDL high-density lipoprotein, LDL low-density lipoprotein, HDLTC ratio the ratio of high-density lipoprotein-globulin to total cholesterol.

To determine whether decreased O_2_ delivery capacity is a gradual, early and common pathogenic feature that occurs with aging, we recruited an independent cohort of 301 human subjects (Supplementary Table S1) ranging in age from 20 to 85 years (168 males, 133 females) and measured functional changes in their RBCs (Fig. 1a). With respect to RBC function, we measured the P50, which is important for assessing the ability of RBCs to release O_2_, by accurately measuring Hb-O_2_ binding affinity. We observed a significant yet gradual decrease in P50 with age (Fig. 1b), which represents increased O_2_ binding affinity and reduced O_2_ release, with no significant differences between males and females (Supplementary Fig. S1a). These results show that reduced RBC O_2_ delivery ability is a common and early feature of the aging process in both sexes and raises the possibility that RBC functional decline (i.e., a reduced ability to deliver O_2_ to peripheral tissues) promotes aging-related tissue functional decline.

To assess this possibility, we conducted linear regression analysis of age and clinical parameters (adjusted for BMI and sex) to identify clinical parameters associated with age-dependent functional decline. We found that 14 clinical characteristics were positively correlated with age, while 11 clinical features were negatively associated with age (Supplementary Fig. S1b). Notably, cardiovascular parameters (increased blood pressure), liver function indicators (elevated AST and TBA levels), renal function indicators (increased blood uric acid and urea levels and a decreased eGFR) and metabolic impairment (increased glucose and triglyceride levels) are clinical parameters commonly associated with age-dependent functional decline (Fig. 1c). Next, to precisely define and compare the interactive links between RBC functional decline and multiple tissue dysfunctions during aging, we used the GSVA R package to calculate the age-related RBC, renal, liver and blood pressure (BP) functional scores (Supplementary Fig. S1c). We observed that RBC and renal functional scores decreased with age, whereas the BP score significantly increased with age (Fig. 1d), which further explains the decrease in tissue function during aging. Further pairwise comparisons of the calculated specific functional scores revealed that the RBC functional score was significantly positively correlated with the renal function score and significantly and negatively correlated with the BP score (Fig. 1e; Supplementary Table S2). Thus, we determined that the gradual decrease in RBC O_2_ release capacity is an early aging-related cellular dysfunction that is strongly linked to the progression of aging-related tissue dysfunction.

### Age-dependent decreases in BPGM-mediated glucose metabolism underlie RBC functional decline

Owing to the lack of transcriptional and translational activity, metabolomics is the most appropriate omics method for identifying changes associated with RBC function during aging. Thus, we conducted high-throughput unsupervised metabolomics analysis of RBCs isolated from human subjects ranging in age from 20 to 85 years. Approximately 537 metabolites were detected in the RBCs (Supplementary Fig. S1d), and the QC samples in the PCA score plot clustered well, with 90% of the metabolites having a coefficient of variation of less than 30%, ensuring the high quality of our RBC metabolomic profiling data (Supplementary Fig. S1e, f). The PLS-DA score plot revealed differences in the metabolic fingerprints during aging (Fig. 1f). We identified 111 metabolites that were positively correlated and 81 metabolites that were negatively correlated with age (Pearson’s correlation, BH-adjusted *P* < 0.05, adjusted for BMI and sex; Supplementary Fig. S1g, Data S1). Pathway enrichment analysis of aging-related metabolites revealed that glycolysis, pentose phosphate pathway (PPP), purine metabolism, and glutathione metabolism were markedly altered during aging (Fig. 1g). These metabolic pathways are closely related to the regulation of O_2_ release, energy balance, and ROS homeostasis in erythrocytes. With respect to glycolysis, we observed significant increases in the levels of glycolytic intermediates, including glucose/fructose-6-phosphate (G6P/F6P), fructose-1,6-bisphosphate (F-1,6-BP), NAD^+^, ATP, pyruvate, and lactate, during aging (Fig. 1h, i; Supplementary Fig. S1h). With respect to the levels of PPP intermediates, including 6-phosphogluconate (6PG), glutathione (GSH), sedoheptulose-7-phosphate (Sed7P), and ribose-1/5-phosphate (R1P/R5P), significantly increased with age (Fig. 1h, i). We also observed an increase in RBC reactive oxygen species (ROS) levels with aging (Fig. 1j). These results suggest that the PPP pathway is significantly activated during aging to counteract the increase in ROS content. Additionally, we observed that the level of 2,3-bisphosphoglycerate (2,3-BPG, a glycolytic metabolite generated by the RBC-specific Rapoport–Luebering shunt (RLS) and a known allosteric modulator that regulates Hb-O_2_ binding affinity^25^) gradually decreased with aging, with a trajectory similar to that of P50 (Fig. 1k). These findings prompted us to measure the activity of BPG mutase (BPGM), the first enzyme in the RLS. BPGM activity was significantly lower in the RBCs of elderly individuals than in those of young individuals (Fig. 1l). These results suggest that despite the increase in RBC glycolysis with aging, the suppression of the RLS accounts for the reduction in 2,3-BPG activity.

Similar to the findings in humans, we observed that the P50 of RBCs in 15-month-old WT mice was significantly lower than that in young 4-month-old WT mice, while the ROS level was significantly greater (Fig. 1m, n). Consistent with RBC human metabolomics signature, metabolomic profiling of mouse RBCs revealed significant differences in the metabolic fingerprints between young and aged mice (Fig. 1o), which was characterized by elevated lactate and pyruvate levels but decreased 2,3-BPG levels in aged mice compared with young WT mice (Fig. 1p; Supplementary Fig. S1i). Moreover, the ratio of GSH to GSSG significantly increased in aged mice (Fig. 1p; Supplementary Fig. S1i). Thus, our data demonstrated that humans and mice experienced similar age-dependent decreases in O_2_ delivery capability and 2,3-BPG activity along with increased glycolysis and PPP activity to combat increased oxidative stress (Fig. 1q).

### RBC-specific genetic ablation of BPG mutase reduces O2 release and accelerates aging

**Fig. 2:**
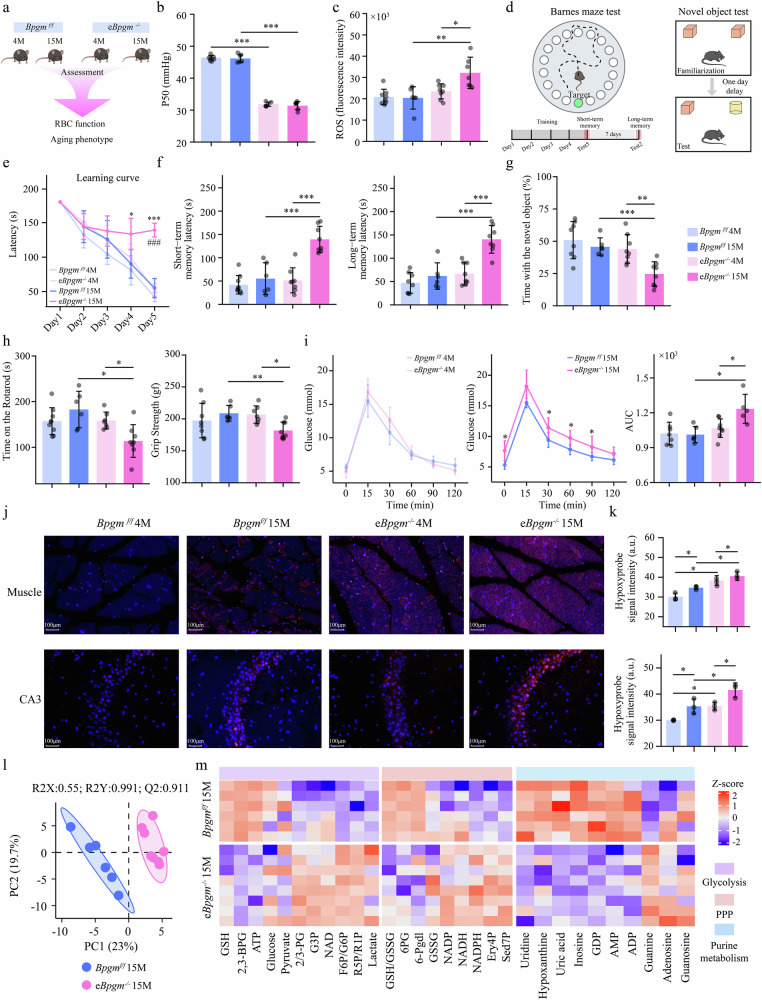
Genetic ablation of BPGM in RBCs accelerates aging. **a** Schematic representation of the experimental design. RBC function (P50 and ROS levels) and aging phenotype (cognitive ability, muscle strength and glucose metabolism) were evaluated in 4-month-old and 15-month-old *Bpgm*^*f/f*^ and *eBpgm*^*–/–*^ mice (males, *n* = 5–8 in each group). **b**, **c** P50 and ROS levels in RBCs from 4-month-old and 15-month-old B *Bpgm*^*f/f*^ and *eBpgm*^*–/–*^ mice. **d** Schematic diagrams of the BM test and NOR test. **e** Learning curve was constructed as the time in seconds (i.e., the latency period) for the mice to find the escape box. *15-month-old *Bpgm*^*f/f*^ mice vs 15-month-old *eBpgm*^*–/–*^ mice; #4-month-old vs 15-month-old *eBpgm*^*–/–*^ mice. **f** Short-term memory and long-term memory were assessed as the time in seconds (latency period) for mice to find the escape box. **g** Percentage of time spent exploring the novel object was quantified in the final trial of the NOR test. **h** Muscle function was measured using rotarod and grip strength tests. The results of the rotarod test are expressed as time in seconds on the rotarod. The results of the grip strength test are expressed as the maximum grip strength value. **i** An intraperitoneal glucose tolerance test was performed to assess glucose metabolism in 4-month-old and 15-month-old *Bpgm*^*f/f*^ and *eBpgm*^*–/–*^ mice. **j** Hypoxyprobe staining of muscle and hippocampal CA3 tissue from *Bpgm*^*f/f*^ and *eBpgm*^*–/–*^ mice. Red reflects the level of oxygen deficiency; the more intense the color is, the greater the degree of oxygen deficiency. **k** Quantification of hypoxia intensity in the muscle and hippocampal CA3 region. **l** PLS-DA score plot of RBC metabolic fingerprints between 15-month-old *Bpgm*^*f/f*^ and *eBpgm*^*–/–*^ mice. **m** Heatmap of glycolysis, the PPP and purine metabolism intermediates in the RBCs of 15-month-old *Bpgm*^*f/f*^ and *eBpgm*^*–/–*^ mice. Data are presented as mean ± SD. ^*^P < 0.05, ^**^P < 0.01, ^***^P < 0.001, and ^###^*P* ^<^ 0.001.

Extending upon our data from human studies, we conducted proof-of-principle genetic studies to determine the essential role of erythrocyte BPGM with advancing age. Briefly, we generated mice with RBC-specific ablation of BPGM (*eBpgm*^*–/–*^ mice) by mating *Bpgm*^*f/f*^ mice with *epoR-Cre* mice (for details, see the Materials and methods) and compared RBC function and aging-related phenotypes, including primarily cognitive ability, muscle function, and glucose tolerance, between the control (*Bpgm*^*f/f*^) and *eBpgm*^*–/–*^ mice at 4 and 15 months of age (Fig. 2a). Western blot analysis confirmed that knockout of BPGM was specific to erythrocytes, as BPGM expression was unchanged in other tissues (Supplementary Fig. S2a). No significant differences in body weight were detected between *Bpgm*^*f/f*^ and *eBpgm*^*–/–*^ mice at 4 and 15 months of age (Supplementary Fig. S2b). The P50 of *eBpgm*^*–/–*^ mice was significantly lower than that of *Bpgm*^*f/f*^ mice at both 4 and 15 months (Fig. 2b), whereas the ROS levels in the RBCs of *eBpgm*^*–/–*^ mice, but not in the RBCs of *Bpgm*^*f/f*^ mice, significantly increased with age, and the ROS levels were significantly greater in the RBCs of 15-month-old *eBpgm*^*–/–*^ mice than *Bpgm*^*f/f*^ mice of the same age (Fig. 2c). These results indicate that genetic ablation of BPGM in RBCs leads to a decrease in O_2_ release and an increase in oxidative stress. Consistent with the findings of a previous study from the Mayo Clinic Experience^26^, RBC counts and Hb and HCT levels were higher in *eBpgm*^*–/–*^ mice than in *Bpgm* ^*f/f*^ mice at both 4 and 15 months of age (Supplementary Table S3), indicating that the genetic ablation of erythrocyte-specific BPGM leads to erythrocytosis as a compensatory response to overcome insufficient O_2_ delivery from mature RBCs.

Next, we applied the Barnes maze (BM) test and novel object recognition (NOR) test to evaluate the differences in cognition (i.e., learning and memory abilities) between *Bpgm*^*f/f*^ and *eBpgm*^*–/–*^ mice (Fig. 2d). Compared with 4-month-old *eBpgm*^*–/–*^ mice, 15-month-old *eBpgm*^*–/–*^ mice took more time to find the escape box on Day 5 following the 4-day training period, but the time needed by *Bpgm*^*f/f*^ mice did not significantly change between 4 and 15 months of age (Fig. 2e). Compared with 4-month-old *eBpgm*^*–/–*^ mice, 15-month-old *eBpgm*^*–/–*^ mice exhibited worse short-term and long-term memory, but the memory of *Bpgm*^*f/f*^ mice did not significantly change during aging (Fig. 2f). Moreover, as *Bpgm*^f/f^ mice aged, they did not show a significant change in terms of exploring new objects, whereas new object exploration by *eBpgm*^*–/–*^ mice significantly decreased. Similar to the BM test results, *eBpgm*^*–/–*^ mice exhibited less interest in investigating novel objects than *Bpgm*^*f/f*^ mice did at 15 months (Fig. 2g). We also assessed muscle function using the rotarod test and grip strength test. Compared with 4-month-old *eBpgm*^*–/–*^ mice, 15-month-old *eBpgm*^*–/–*^ mice had a reduced ability to remain on the rotarod, while there was no significant difference in duration on the rotarod between 15-month-old and 4-month-old *Bpgm*^*f/f*^ mice (Fig. 2h). The grip strength test revealed that the 15-month-old *eBpgm*^*–/–*^ mice displayed significantly lower grip strength than age-matched *Bpgm*^*f/f*^ mice (Fig. 2h). Additionally, we analyzed glucose homeostasis in the mice using an intraperitoneal glucose tolerance test. We observed that the ability of *Bpgm*^*f/f*^ mice to metabolize glucose did not change significantly between 4 and 15 months of age, whereas the ability of *eBpgm*^*–/–*^ mice to metabolize glucose decreased significantly with age (Fig. 2i). Moreover, glucose metabolism by 4-month-old *Bpgm*^*f/f*^ and *eBpgm*^*–/–*^ mice did not differ, but compared with age-matched *Bpgm*^*f/f*^ mice, 15-month-old *eBpgm*^*–/–*^ mice metabolized glucose less efficiently (Fig. 2i**)**. Additionally, the hypoxyprobe staining results indicated that hypoxia in the muscles and hippocampal CA3 region significantly increased in *eBpgm*^*–/–*^ and *Bpgm*^*f/f*^ mice with age, that hypoxia in the muscles and hippocampal CA3 sections was significantly greater in 4- and 15-month-old *eBpgm*^*–/–*^ mice than that in age-matched *Bpgm*^*f/f*^ mice (Fig. 2j, k), and that there were no significant differences in the levels of hypoxia in the hippocampal CA1 and DG regions between *eBpgm*^*–/–*^ and *Bpgm*^*f/f*^ mice (Fig. S2c). Research has shown that the CA3 region of the hippocampus is closely related to learning and memory ability^27^. Finally, metabolomics analysis of RBCs between 15-month-old *eBpgm*^*–/–*^ and *Bpgm*^*f/f*^ mice was performed to evaluate differences in key aging-related metabolites. We observed significant differences in the RBC metabolic fingerprints between 15-month-old *eBpgm*^*–/–*^ and *Bpgm*^*f/f*^ mice of the same age (Fig. 2l). In particular, compared with those in 15-month-old *Bpgm*^*f/f*^ mice, the 2,3-BPG, ATP, 6PG levels and the ratio of GSH to GSSG in the RBCs of *eBpgm*^*–/–*^ mice were significantly lower (Fig. 2m), indicating that the antioxidant stress response in and O_2_ release capacity of 15-month-old *eBpgm*^*–/–*^ mice were reduced. In addition, the levels of inosine in RBCs were significantly lower in 15-month-old *eBpgm*^*–/–*^ mice than in 15-month-old *Bpgm*^*f/f*^ mice, whereas the Sed7P, Ery4P and R5P/R1P levels were significantly greater in 15-month-old *eBpgm*^*–/–*^ mice than 15-month-old *Bpgm*^*f/f*^ mice (Fig. 2m). These results suggest that inosine metabolism and the nonoxidative pathway of PPP are more active in 15-month-old *eBpgm*^*–/–*^ mice than in 15-month-old *Bpgm*^*f/f*^ mice, similar to the results observed in aging humans. Taken together, our findings provide genetic evidence that decreased BPGM-mediated 2,3-BPG production in erythrocytes underlies aging-induced erythrocyte glucose metabolic impairment and functional decline and drives peripheral tissue hypoxia and dysfunction with aging.

### Increased PNP activity induces inosine-derived R1P as a compensatory fuel for RBCs in elderly individuals

**Fig. 3:**
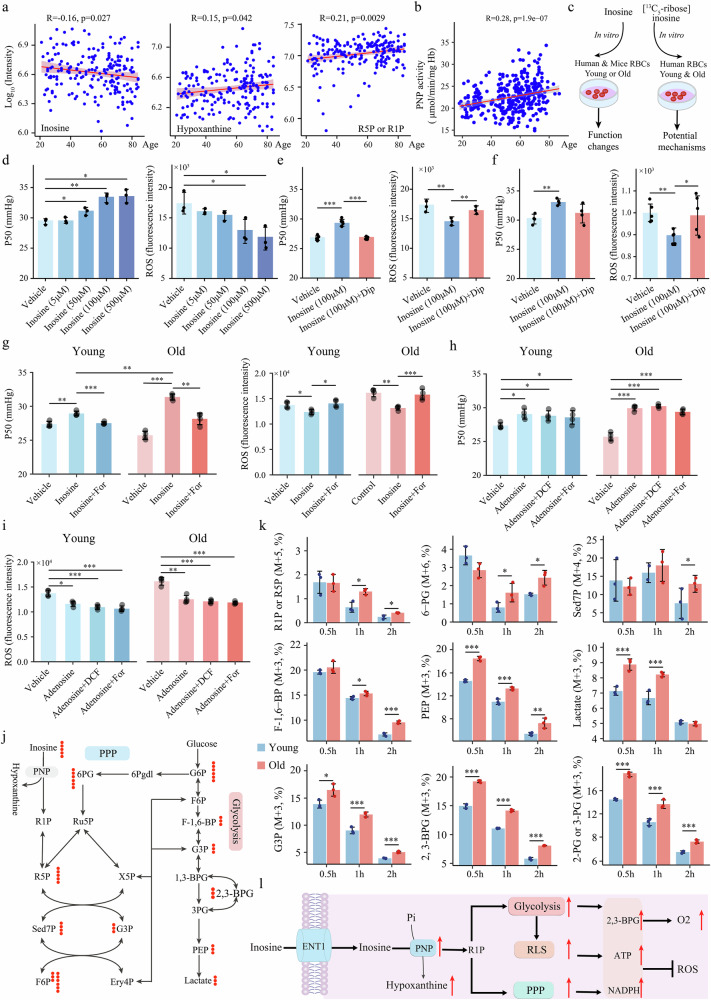
The eENT1-PNP axis provides inosine-derived R1P as a compensatory fuel to improve RBC function and combat aging. **a** Changes in the concentrations of inosine, hypoxanthine and R1P/R5P in RBCs with age. **b** Changes in PNP enzyme activity in RBCs as a function of age. **c** Flowchart of inosine treatment and functional analysis of RBCs from humans and mice. **d** Changes in the P50 and ROS levels following treatment of human erythrocytes with different inosine concentrations for 1 h. **e** Changes in the P50 and ROS levels following incubation of human erythrocytes (28 years) with 100 µM inosine for one hour in the absence or presence of dipyridamole (DIP, an ENT1 inhibitor). **f** Changes in the P50 and ROS levels following incubation of RBCs from 2-month-old mice with 100 µM inosine in the absence or presence of dipyridamole. Data are presented as mean ± SD. *n* = 4–6 in each group. **g** Changes in the P50 and ROS levels following incubation of human RBCs with 100 µM inosine for 1 h in the absence or presence of forodesine (For, a PNP inhibitor); the RBCs were obtained from four younger individuals (*n* = 4, 30 years) and four older individuals (*n* = 4, 71 years). **h,** **i** Changes in the P50 and ROS levels following the incubation of human RBCs with 100 µM adenosine for 1 h in the absence or presence of forodesine and 2’-deoxycoformycin (DCF, an ADA inhibitor); the RBCs were obtained from four younger individuals (*n* = 4, 30 years) and four older individuals (*n* = 4, 71 years). **j** Schematic diagram of inosine metabolism in RBCs. Inosine metabolic flux analysis was used to determine inosine metabolism in RBCs during aging. The RBCs were obtained from three young individuals (*n* = 3, 30 years) and three elderly individuals (*n* = 3, 71 years) and harvested at three time points (0.5, 1, 2 h) for metabolomics flux analysis. The red dots represent the number of ^13^C-labeled carbon atoms in the inosine metabolic flux analysis experiments. **k** RBCs were incubated with [^13^C_5_]inosine for 0.5, 1 or 2 h, and the labeled metabolites related to glycolysis and the PPP are plotted. **l** Depiction of inosine-promoted RBC glucose metabolism. As aging progresses, increased PNP activity drives increased inosine degradation, thereby producing R1P, which enters glucose metabolism via PPP to increase glycolysis, increasing the production of 2,3-BPG and ATP. These results suggest that inosine can improve oxygen release by and the oxidative stress resistance of RBCs. The arrow represents the elderly group vs the young group; the red arrow indicates that metabolic pathway activity is greater in the elderly group than in the young group. Data are presented as mean ± SD. ^*^P < 0.05, ^**^P < 0.01, ^***^P < 0.001.

In addition to the glucose metabolic reprogramming observed in RBCs during aging, our metabolomics results revealed that purine metabolism in RBCs was also significantly impacted during aging (Fig. 1g). Specifically, the level of inosine in RBCs significantly decreased with age (Fig. 3a), similar to the gradual decreases in the 2,3-BPG level and P50. The decrease in inosine content in RBCs as a function of age was accompanied by an increase in hypoxanthine and ribose-1/5-phosphate (R1P/R5P) (Fig. 3a). Similarly, we detected significant changes in purine metabolism in aged mice. In particular, the levels of hypoxanthine and R1P/R5P were significantly increased in aged mice compared with young mice (Supplementary Fig. S3b), indicating inosine catabolism increases with age in mice. These findings prompted us to measure the activity of PNP, the enzyme that catalyzes the conversion of inosine to hypoxanthine and R1P. The results revealed that PNP activity gradually increased during aging (Fig. 3b). Our results demonstrated that increases in PNP activity and inosine catabolism to increase R1P/R5P levels are common features of aging in both humans and mice. Early studies reported that the ribose produced from inosine degradation is an important carbon source for the body, which can undergo glycolysis to provide ATP or enter the pentose phosphate pathway^28^. Thus, a gradual increase in RBC PNP activity with age contributes to the aging-dependent catabolism of inosine, indicating that inosine-derived ribose may be an additional fuel involved in the enhanced glycolysis and the PPP pathway activity in the RBCs of elderly individuals.

To address this possibility, we conducted in vitro experiments in which RBCs from 28-year-old human volunteers and 2-month-old mice were incubated with inosine to determine the effects of inosine on RBC function (Fig. 3c). We observed that inosine significantly increased the P50 of cultured human RBCs in a dose-dependent manner within 1 h, peaking at 100 μmol (Fig. 3d). Moreover, inosine significantly reduced the ROS levels in cultured human RBCs (Fig. 3d). Given that ENT1 is the major transporter for inosine uptake in RBCs^29^, we hypothesized that ENT1 is a potential candidate for the uptake of inosine. To test this hypothesis, we treated cultured primary human and murine RBCs with a specific ENT inhibitor in the presence of inosine. We found that the inosine-mediated increase in P50 and decrease in ROS levels were significantly blocked by dipyridamole (DIP, a specific ENT inhibitor) in both cultured human and murine RBCs (Fig. 3e, f). Thus, we provide both human and murine evidence that inosine enters RBCs through the ENT1 transporter, directly increasing O_2_ release and reducing ROS levels. Next, given that PNP is a key enzyme that drives inosine catabolism and on the basis of our finding that PNP activity increases during aging, we determined the importance of PNP in the ENT1-dependent uptake of inosine-mediated increased O_2_ delivery and anti-oxidative stress capability, in primary cultured RBCs from young (*n* = 4, 30 years) and elderly individuals (*n* = 4, 71 years) in the presence of PNP inhibitors. Intriguingly, forodesine (For, a specific PNP inhibitor) significantly attenuated the inosine-mediated increase in P50 and decrease in ROS levels in cultured RBCs from both young and elderly individuals (Fig. 3g). Notably, the P50 induced by inosine in RBCs from elderly individuals was greater than that in RBCs from young individuals, which further supports our findings that elderly individuals have increased RBC PNP enzyme activity (Fig. 3g).

Moreover, early studies showed that adenosine promotes RBC function via activation of the A2B receptor (ADORA2B)^30^_,_ and in view of the fact that adenosine can be converted to inosine via adenosine deaminase (ADA) catalysis, we were prompted to further delineate whether inosine-induced RBC function is dependent on adenosine generated by ADA signaling. In contrast to the gradual increase in PNP activity (Fig. 3b), there were no significant changes in ADA activity during aging (Supplementary Fig. S3c). Moreover, adenosine treatment directly increased P50 and reduced ROS levels in cultured primary RBCs from both young and elderly individuals, whereas neither forodesine nor 2’-deoxycoformycin (DCF, an ADA inhibitor) abolished these adenosine-mediated effects (Fig. 3h, i), ruling out the possibility that the adenosine-mediated increase in RBC function is dependent on the activity of ADA or PNP. In contrast, adenosine-induced functional changes in RBCs, including changes in the P50 and anti-ROS capability, were significantly reduced in cultured primary RBCs from WT mice treated with PSB1115 (an ADORA2B inhibitor) and in RBCs from *Adora2b*^*–/–*^ mice (Supplementary Fig. S3d, e). These results indicate that adenosine primarily improves the P50 and ROS levels in erythrocytes through ADORA2B, providing both pharmacological and genetic evidence that adenosine exerts its effects through ADORA2B in RBCs. Thus, we demonstrated that the regulatory effects of adenosine and inosine on erythrocyte function differ: adenosine functions through the A2B receptor, whereas inosine acts through PNP-mediated inosine metabolism.

Next, to directly elucidate how inosine is metabolized inside RBCs during aging, [^13^C_5_-ribose]-inosine metabolic flux tracing analysis was performed. Briefly, we cultured primary human RBCs isolated from young (*n* = 3, 30 years) and elderly (*n* = 3, 71 years) individuals in the presence of isotopically labeled inosine and unlabeled glucose for up to 2 h, followed by flux analyses to determine the fate of the inosine-derived ^13^C_5_-ribose (Fig. 3j; Supplementary Data S2). Initially, we observed that the [^13^C_5_]-inosine concentration in the supernatant was significantly lower in the elderly group than in the young group after 0.5–1 h of incubation (Supplementary Fig. S3f), whereas the level of inosine in the RBCs did not significantly differ (Supplementary Fig. S3g); however, the levels of [^13^C_5_]-R1P/R5P were significantly higher in RBCs from the elderly group than in RBCs from the young group after 1–2 h of incubation (Fig. 3k). These findings are consistent with the increased activity of PNP in RBCs from elderly individuals (Fig. 3b), which converts [^13^C_5_]-inosine to [^13^C_5_]-R1P. Further tracing analyses revealed that inosine-derived R1P was metabolized into glycolysis intermediates, with significantly higher levels of [^13^C_3_]-F-1,6-BP, [^13^C_3_]-G3P, [^13^C_3_]-2-PG/3-PG, [^13^C_3_]-PEP and [^13^C_3_]-lactate in RBCs from older individuals (Fig. 3k). Inosine-derived R1P also fueled the expansion of the PPP in RBCs from the elderly group, as evidenced by the increased incorporation of [^13^C_6_] into 6-PG and [^13^C_4_]-Sed7P (Fig. 3k). Finally, we found that the increase in the level of inosine-derived R1P/R5P also resulted in increased incorporation of R1P-derived ^13^C into 2,3-BPG in RBCs from the elderly group (Fig. 3k). Overall, we revealed that the ENT1-mediated uptake of inosine coupled with increased PNP activity-induced inosine-derived R1P production is an additional mechanism that counteracts age-related glucose metabolism impairment as a compensatory mechanism by promoting glycolysis, PPP and even RLS in the RBCs of elderly individuals (Fig. 3l).

### eENT1-dependent inosine uptake is essential for promoting RBC function and combating aging

**Fig. 4:**
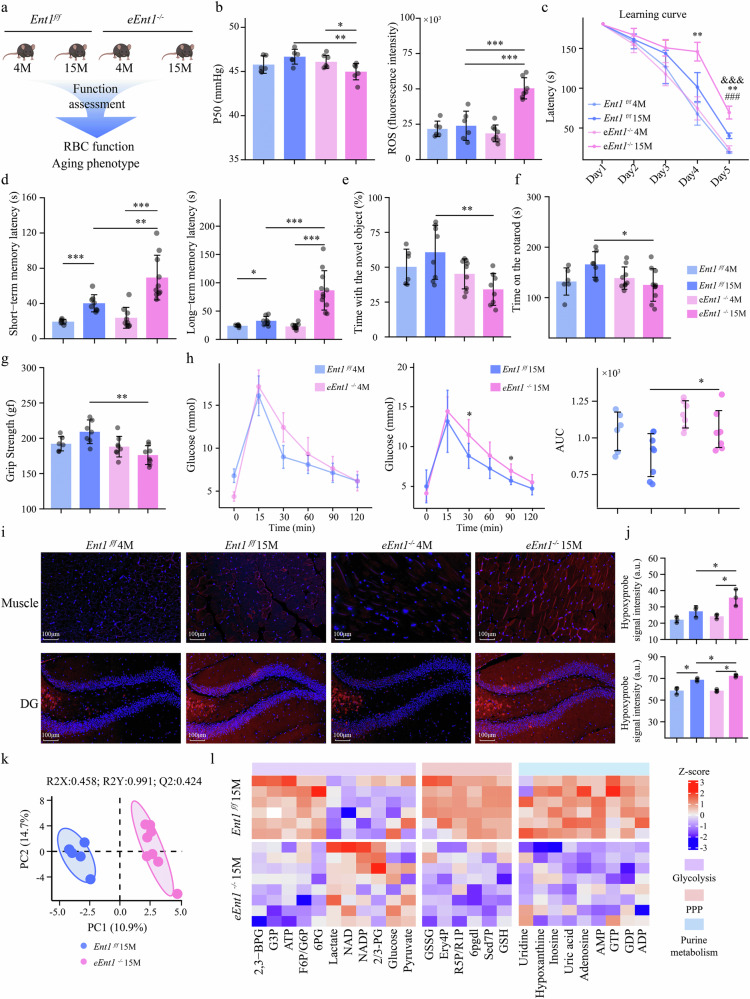
Genetic ablation of ENT1 in RBCs accelerates aging. **a** Schematic representation of the experimental design. The aging phenotypes (cognitive ability, muscle strength and glucose metabolism) and RBC function of 4-month-old and 15-month-old *eEnt1*^*–/–*^ and *Ent1*^*f/f*^ mice were evaluated (males, *n* = 5–10 in each group). **b** Changes in the P50 and ROS levels in RBCs from 4-month-old and 15-month-old *Ent1*^f/f^ and *eEnt1*^*–/–*^ mice. **c** Learning curve was constructed as the time in seconds (latency period) for the mice to find the escape box. *15-month-old *Ent1*^*f/f*^ mice vs 15-month-old *eEnt1*^*–/–*^ mice, ^#^4-month-old vs 15-month-old *eEnt1*^*–/–*^ mice, ^&^4-month-old vs 15-month-old *Ent1*^*f/f*^ mice. **d** Short-term memory and long-term memory were measured as the time in seconds (latency period) for mice to find the escape box. **e** Percentage of time spent exploring the novel object was quantified in final trial of NOR test. **f**, **g** Muscle function was measured using rotarod and grip strength tests. The results of the rotarod test are expressed as time in seconds on the rotarod. The results of the grip strength test are expressed as maximum grip strength value. **h** An intraperitoneal glucose tolerance test was performed to assess glucose metabolism capacity among 4-month-old and 15-month-old *Ent1*^*f/f*^ and *eEnt1*^*–/–*^ mice. **i** Hypoxyprobe staining of muscle and DG tissue from *Ent1*^*f/f*^ and *eEnt1*^*–/–*^ mice. Red reflects the level of oxygen deficiency; the more intense the color is, the greater the degree of oxygen deficiency. **j** Quantification of hypoxia intensity in the muscle and hippocampal DG. **k** PLS-DA score plot of RBC metabolic fingerprints between 15-month-old *Ent1*^f/f^ and *eEnt1*^*–/–*^ mice. **l** Heatmap of glycolysis, the PPP and purine metabolism intermediates in the RBCs of 15-month-old *Ent1*^*f/f*^ and *eEnt1*^*–/–*^ mice. Data are presented as mean ± SD. ^*^P < 0.05, ^**^P < 0.01, ^***^P < 0.001, ^###^*P* < 0.001.

To assess the importance of ENT1 in RBC function and its contribution to peripheral tissue hypoxia and functional decline during aging, we generated erythrocyte-specific ENT1-deficient mice (*eEnt1*^*–/–*^) and assessed its effects on O_2_ release and aging-related phenotypes (Fig. 4a). First, complete blood count tests revealed that compared with *Ent1*^*f/f*^ mice, *eEnt1*^*–/–*^ mice had lower RBC counts and Hb and HCT levels at 15 months, whereas the MCV were larger than that of *Ent1*^*f/f*^ mice (Supplementary Table S4), indicating that 15-month-old *eEnt1*^*–/–*^ mice exhibited an anemic phenotype, which is consistent with previous reports^31^. Next, we observed that *eEnt1*^*–/–*^ mice exhibited significantly reduced O_2_ release and significantly increased ROS levels during aging, whereas no significant difference was detected between 4- and 15-month-old *Ent1*^*f/f*^ mice. At 15 months of age, *eEnt1*^*–/–*^ mice had significantly lower P50 values than *Ent1*^*f/f*^ mice did, while ROS levels were significantly higher in *eEnt1*^*–/–*^ mice than in *Ent1*^*f/f*^ mice (Fig. 4b). Additionally, inosine failed to induce O_2_ delivery and anti-ROS capacity in RBCs from 4-month-old *eEnt1*^*–/–*^ mice (Supplementary Fig. S4a), providing direct evidence that eENT1 is essential for the uptake of inosine and its subsequent effects on improving RBC O_2_ delivery and anti-ROS capability.

Finally, we compared the cognitive function of young (4-month-old) and aged (15-month-old) male *Ent1*^*f/f*^ and *eEnt1*^*–/–*^ mice by performing the BM test and novel object test. The results of the BM test revealed that compared with 4-month-old *eEnt1*^*–/–*^ and *Ent1*^*f/f*^ mice, 15-month-old *eEnt1*^*–/–*^ and *Ent1*^*f/f*^ mice took more time to find the escape exit, whereas at 4 months of age, *eEnt1*^*–/–*^ and *Ent1*^*f/f*^ mice did not show significant differences in the time taken to find the escape exit (Fig. 4c). However, at 15 months of age, compared with age-matched controls, *eEnt1*^*–/–*^ mice took significantly more time to find the escape exit on Days 3 and 4 of the training period (Fig. 4c). Short-term memory and long-term memory were significantly reduced in aged *eEnt1*^*–/–*^ and *Ent1*^*f/f*^ mice, whereas compared with age-matched *Ent1*^*f/f*^ mice, 15-month-old *eEnt1*^*–/–*^ mice exhibited worse short-term memory and long-term memory (Fig. 4d). The NOR test results revealed that the interest of *eEnt1*^*–/–*^ and *Ent1*^*f/f*^ mice in exploring new objects did not significantly differ at 4 months of age, but at 15 months, *eEnt1*^*–/–*^ mice demonstrated significantly less interest in exploring new objects than age-matched *Ent1*^*f/f*^ mice did (Fig. 4e). Furthermore, the rotarod test and grip strength test were performed to assess muscle function. Compared with 15-month-old *Ent1*^*f/f*^ mice, 15-month-old *eEnt1*^*–/–*^ mice spent less time on the rotarod, but the difference was not significant at 4 months of age (Fig. 4f). The grip strength tests revealed that the grip strength of 15-month-old *eEnt1*^*–/–*^ mice was significantly worse than that of age-matched *Ent1*^*f/f*^ mice (Fig. 4g). No difference was observed among 4-month-old mice. A glucose tolerance test revealed that the ability of *Ent1*^*f/f*^ mice and *eEnt1*^*–/–*^ mice to metabolize glucose did not significantly differ at 4 months of age (Fig. 4h). Moreover, the glucose metabolism of *Ent1*^f/f^ mice did not significantly change with age (Fig. 4h). However, compared with 4-month-old *eEnt1*^*–/–*^ mice, 15-month-old *eEnt1*^*–/–*^ mice metabolized glucose less efficiently (Fig. 4h). Further tissue staining with a hypoxia probe revealed that hypoxia in the muscles and hippocampal DG, CA1, and CA3 regions significantly increased with age in both *Ent1*^*f/f*^ and *eEnt1*^*–/–*^ mice, whereas 15-month-old *eEnt1*^*–/–*^ mice exhibited more severe hypoxia in the muscles and hippocampal DG region than *Ent1*^*f/f*^ mice did (Fig. 4i, j; Supplementary Fig. S4b). Additionally, metabolomic analysis revealed significant differences in RBC metabolic fingerprints between 15-month-old *eEnt1*^*–/–*^ and *Ent1*^*f/f*^ mice (Fig. 4k). The levels of 2,3-BPG, ATP, 6PG and GSH in the RBCs of 15-month-old *eEnt1*^*–/–*^ mice were significantly lower than those of 15-month-old *Ent1*^*f/f*^ mice (Fig. 4l), indicating that the O_2_ release capacity and anti-oxidative stress capability of 15-month-old *eEnt1*^*–/–*^ mice were impaired. Furthermore, the levels of inosine, hypoxanthine, Sed7P, Ery4P and R5P/R1P in the RBCs of 15-month-old *eEnt1*^*–/–*^ mice were significantly lower than those in the RBCs of 15-month-old *Ent1*^*f/f*^ mice (Fig. 4l). These results suggest that inosine metabolism and the nonoxidative pathway of the PPP activity are lower in 15-month-old *eEnt1*^*–/–*^ mice than in 15-month-old *Ent1*^*f/f*^ mice, similar to the results observed in aging humans. Thus, we provided genetic evidence that RBC ENT1 is essential for counteracting the age-dependent decline in RBC function (i.e., O_2_ release), cognitive ability, muscle function and glucose and inosine metabolism.

### Preclinical studies: Inosine supplementation improves aging-related multifunctional decline

**Fig. 5:**
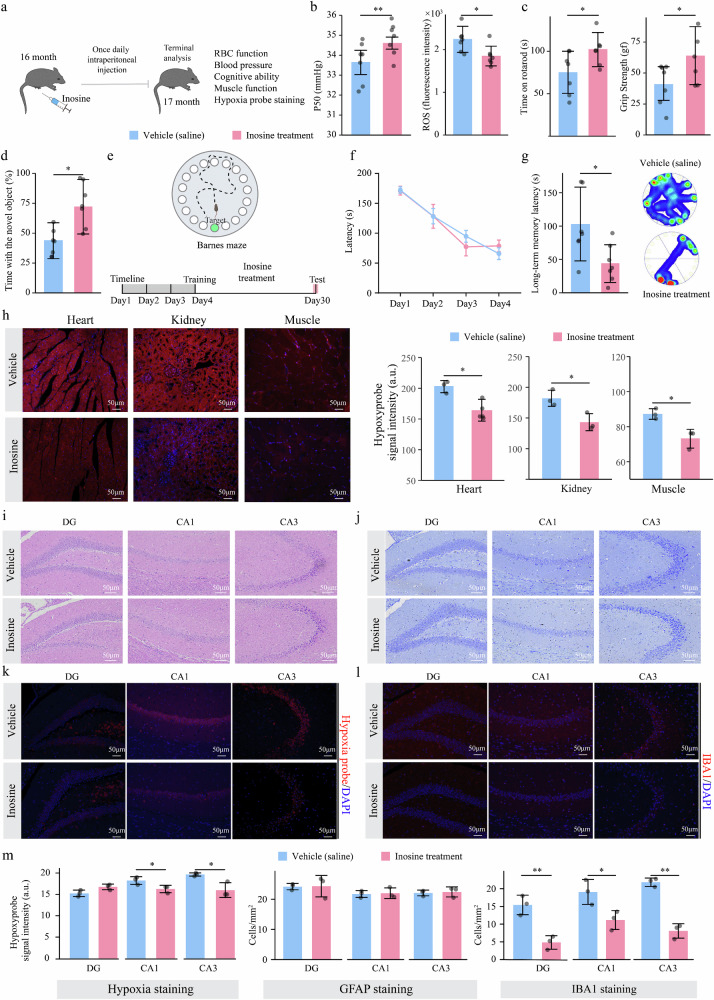
Inosine supplementation impedes age-related RBC and tissue functional decline. **a** Framework for inosine intervention experiments in 16-month-old mice. **b** Differences in the P50 and ROS levels in RBCs between the vehicle- and inosine-treated groups. **c** Rotarod and grip strength tests were performed to assess to muscle function in the control and inosine-treated groups. The gf value represents the maximum grip strength in the grip strength tests. **d** NOR test results for the control and inosine-treated groups. **e** Schematic diagram of the BM test. **f** Learning curve for 4 consecutive days in the BM showing control mice versus inosine-treated mice. Latency refers to the time in seconds needed to find the escape route. **g** The long-term memory of the mice and a heatmap of the movement trajectories in the BM experiment. The simpler the trajectory heatmap is, the faster the target area was found. Latency refers to the time in seconds needed to find the escape route. **h** Hypoxyprobe staining of heart, kidney and muscle. Red reflects the level of oxygen deficiency; the more intense the color is, the greater the degree of oxygen deficiency. **i,** **j** Nissl and HE staining images of the hippocampus (DG; CA1; CA3). **k** Hypoxyprobe staining of different regions of the hippocampus (DG, CA1, and CA3). **l** Immunofluorescence staining of Iba1 in different regions of the hippocampus. Immunofluorescence staining of GFAP is shown in Supplementary Fig. S6. **m** Quantification of hypoxia intensity and astrocyte and microglia cell numbers. Data are presented as mean ± SD of each group. *n* = 3–7 in each group; ^*^P < 0.05, ^**^P < 0.01.

Extending upon the results from our genetic studies, we conducted preclinical studies in WT mice to determine whether inosine supplementation can combat the aging-related decreases in RBC, vascular, muscular, and cognitive function and general tissue hypoxia. Specifically, 16-month-old mice were injected intraperitoneally with inosine every day for 4 weeks, and their P50, blood pressure, body weight, muscle strength and cognitive function were monitored (Fig. 5a). Compared with those in the vehicle group, the RBCs in the inosine-injected group showed an increase in the P50 and a reduction in ROS levels (Fig. 5b), but the blood pressure and weight were not significantly different (Supplementary Fig. S5a, b). Thus, this in vivo preclinical study provides evidence that inosine increases O_2_ release capacity and reduces ROS levels in the RBCs of 16-month-old mice.

Aging is accompanied by a progressive loss of muscle strength^32^. In this study, forelimb grip strength was measured in naturally aged animals with or without inosine treatment (Fig. 5c). Compared with vehicle-injected age-matched control mice, 16-month-old mice treated with inosine exhibited improved forelimb grip strength. Additionally, as shown in Fig. 5c, inosine treatment improved the performance of aged mice in the rotarod test, as they remained on the rotarod significantly longer. Finally, to explore the effects of inosine on spatial recognition and memory, control and inosine-injected mice were subjected to the BM test and NOR test. Compared with age-matched controls, inosine-treated mice were more interested in investigating novel objects (Fig. 5d). Spatial learning and memory were assessed using the BM test (Fig. 5e). Compared with the vehicle-injected control group, the inosine-treated group took significantly less time to find the escape box (Fig. 5f, g). Additionally, we found that the level of hypoxia in the hearts, kidneys, and muscles of the inosine-treated mice was significantly reduced (Fig. 5h). Thus, we provide preclinical evidence that inosine supplementation improves the aging-related decreases in cognitive function and muscle strength and reduces the onset of tissue hypoxia.

Finally, to elucidate the potential cellular effects in the brain mediated by inosine treatment, we conducted H&E staining, hypoxia probe analysis, and immunostaining of the hippocampus, a major region of the brain for learning processing and long-term memory storage. H&E staining of tissue from saline-treated mice revealed extensive necrosis in three regions of the hippocampus accompanied by pyknosis (Fig. 5i). In contrast, inosine treatment reduced pyknosis and necrosis in brain tissue. Additionally, Nissl staining revealed that, compared with saline treatment, inosine treatment increased the density of neurons in the dentate gyrus (DG) and cornu ammonis (CA1 and CA3) regions of the hippocampus (Fig. 5j). The hypoxyprobe staining results of the DG and CA1 and CA3 regions revealed that the staining intensity of the inosine-treated group was significantly lower than that of the saline-treated group (Fig. 5k, m). GFAP staining revealed no obvious difference in astrocyte antigen expression between the saline-treated and inosine-treated groups (Supplementary Fig. S5c; Fig. 5m). We then evaluated the changes in the number and morphology of microglia through Iba1 antibody staining in the DG and CA1 and CA3 regions of the hippocampus (Fig. 5l, m). Compared with those in the saline-treated group, the number of microglia in the inosine-treated group was significantly lower, and more branches were observed, indicating a more active morphology (Fig. 5k, l). Taken together, the results of our preclinical studies demonstrate that inosine treatment promotes O_2_ release from and reduces ROS levels in RBCs; counteracts age-related heart, kidney, muscle, and hippocampus hypoxia, inflammation, and neuron degeneration; and improves cognitive ability and muscle strength.

### 2,3-BPG competes with Pi to bind to the phosphate-binding domain of PNP as an endogenous inhibitor

**Fig. 6:**
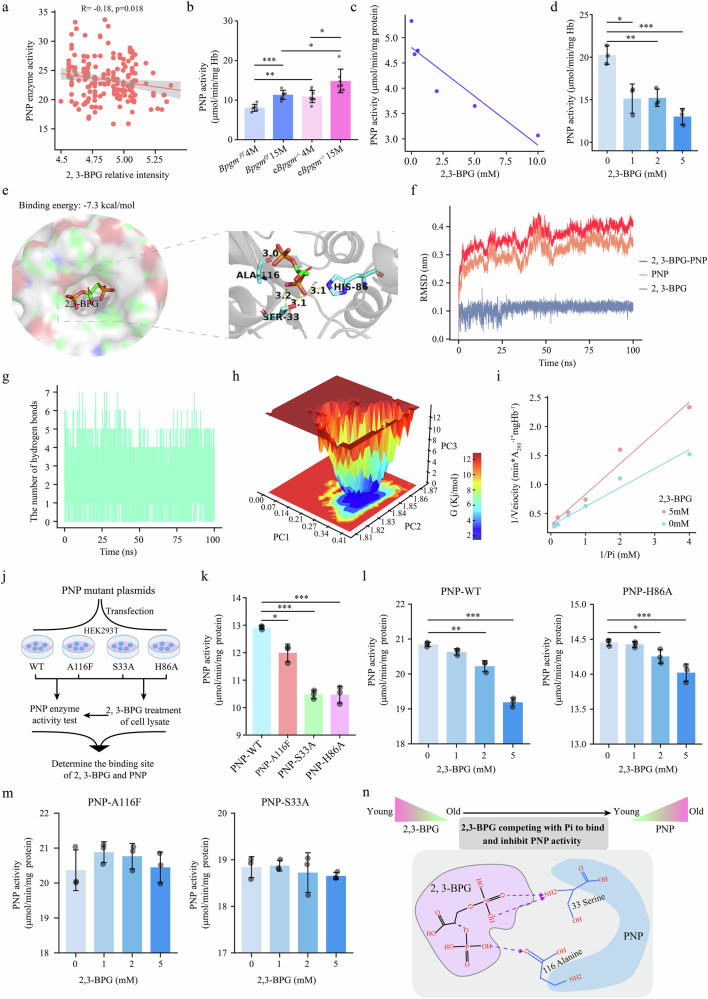
2,3-BPG affects the degradation of inosine in RBCs by inhibiting PNP activity. **a** Correlations between RBC PNP enzyme activity and 2,3-BPG concentration in aging populations. **b** PNP enzyme activity in RBCs from 4-month-old and 15-month-old *Bpgm*^*f/f*^ and *eBpgm*^*–/–*^ mice. **c** The inhibition of PNP by 2,3-BPG. The concentrations of the substrates inosine and phosphate were 0.1 mM and 1 mM, respectively. In addition to PNP, the assays contained 100 mM Tris/HCl (pH = 7.5), 0.05 U/mL xanthine oxidase, various amounts of 2,3-BPG, and 2 μL of PNP (0.1 mg/mL) in a final volume of 0.2 mL with incubation at 30 °C. **d** Effect of 2,3-BPG on PNP activity in RBC lysates from 4-month-old *eBpgm*^*–/–*^ mice. The concentrations of the substrates inosine and phosphate were 0.1 mM and 0.1 mM, respectively, and each sample contained 2 μL of RBC lysate (1 μL of red blood cells added to 2 μL of lysis buffer). The other assay conditions were as described in **c**. **e** Molecular docking of 2,3-BPG with PNP. **f** RMSD curves of the 2,3-BPG-PNP complexes indicating the structural stability of the complexes. **g** The number of hydrogen bonds reflects the strength of 2,3-BPG-PNP complex binding. **h** Free energy distribution diagrams indicating the conformations of the 2,3-BPG-PNP complexes with the least energy throughout the simulation. If the complex interaction is weak or unstable, multiple rough minimum energy clusters are present in the free-energy distribution; in contrast, strong and stable interactions can lead to the formation of nearly single and smooth energy clusters in the potential energy distribution. **i** Effect of 2,3-BPG on the activity of PNP in RBC lysates from 4-month-old *eBPGM*^*–/–*^ mice treated with phosphate as the variable substrate. The concentration of the substrate inosine was 0.1 mM, and each sample contained 2 μL of RBC lysate. The other assay conditions were as described in (**c**). **j** The interactions between 2,3-BPG and the PNP site were determined through transfection of a PNP plasmid into 293 T cells and subsequent PNP activity measurement. **k** Changes in PNP enzyme activity in 293 T cells transfected with plasmids with different PNP mutations. PNP: Control, A116F, alanine 116 mutated to phenylalanine; S33A: serine 33 mutated to alanine; and H86A, histidine 86 mutated to alanine. **l**, **m** Effects of 2,3-BPG on the enzyme activity of different PNP mutants. **n** Summary of the interactions between 2,3-BPG and PNP (arrows indicate the interaction sites between 2,3-BPG and PNP). 2,3-BPG competes with Pi to bind to the 33-serine and 116-alanine residues of PNP and inhibits PNP activity. Data are presented as mean ± SD. *P < 0.05, **P < 0.01, ***P < 0.001.

Given our findings that increased PNP activity-mediated R1P production from inosine is an important and compensatory mechanism for maintaining RBC bioenergetics during aging, we aimed to define the molecular basis underlying PNP activation during aging. Therefore, we revisited our metabolomics profiling results and conducted correlation studies of PNP activity with all the significantly altered metabolites during aging. Intriguingly, we found that the 2,3-BPG level was significantly negatively correlated with PNP activity during aging in humans (Fig. 6a), suggesting a new but compelling possibility that 2,3-BPG, as a small metabolite, inhibits PNP activity. Extending upon the results from these correlation studies in humans, we took advantage of our erythrocyte-specific BPGM knockout mice *(eBpgm*^*–/–*^*)*. Like that in humans, PNP activity was significantly elevated in RBCs of 15-month-old *Bpgm*^*f/f*^ mice compared with those of 4-month-old young mice. Surprisingly, we observed significantly higher PNP activity in *eBpgm*^*–/–*^ mice than in *Bpgm*^*f/f*^ control mice at 4 months of age and a further increase in PNP activity in *eBpgm*^*–/–*^ mice at 15 months of age compared with the controls (Fig. 6b). Thus, we concluded that compared with control mice, mice lacking the ability to produce 2,3-BPG in their RBCs had greater PNP activity at both 4 and 15 months of age, immediately suggesting that 2,3-BPG is likely a metabolite that can inhibit PNP activity in RBCs.

To determine whether 2,3-BPG can directly inhibit PNP activity, we conducted an in vitro assay to quantify the PNP activity in the absence and presence of different physiologically relevant concentrations of 2,3-BPG. The results (Fig. 6c) revealed that 2,3-BPG inhibited PNP activity in a concentration-dependent manner. Next, to determine whether 2,3-BPG inhibits PNP under more physiological conditions, we prepared RBC extracts from 4-month-old *eBpgm*^*–/–*^ mice and measured PNP activity in the presence of different concentrations of 2,3-BPG. The results (Fig. 6d) revealed that 2,3-BPG inhibited PNP activity in RBC extracts in a dose-dependent manner. Thus, we demonstrated that 2,3-BPG directly inhibits PNP activity in both a purified PNP system and RBC extracts within the concentration range normally found in RBCs.

Having demonstrated the inhibitory effect of 2,3-BPG on PNP activity, we sought to determine the underlying molecular mechanism. First, we performed a molecular docking analysis to assess the potential binding between PNP and 2,3-BPG. The results revealed that 2,3-BPG interacted with a cavity on the protein surface with a binding energy of –7.3 kcal/mol, indicating the high binding affinity of 2,3-BPG to PNP (Fig. 6e). Next, we examined the interactions of the phosphate groups of 2,3-BPG, which formed four hydrogen bonds with the Ala116, Ser33, and His86 residues of PNP (Fig. 6e). PNP active site prediction analysis revealed that the predicted active pocket of PNP contains the Ala116, Ser33, and His86 residues (Supplementary Fig. S6a), further supporting the possibility that 2,3-BPG inhibits PNP activity by binding to its active pocket. Thus, we performed molecular dynamics simulations to determine the binding stability between 2,3-BPG and the active site of human PNP. The root mean square deviation (RMSD) values are often used to determine the stability of protein–ligand complexes, with a smaller RMSD value representing a more stable complex^33^. Thus, quantification of the RMSD values by constructing RMSD curves revealed that the curves of the 2,3-BPG and PNP complexes nearly overlapped, fluctuating within 0.5 nm and stabilizing at 0.38 ± 0.15 nm, indicating the strong binding stability of 2,3-BPG to the PNP protein (Fig. 6f). Given that the number of hydrogen bonds reflects the strength of protein–ligand binding^34^, the results of the atomic analyses further supported the high binding affinity between 2,3-BPG and PNP owing to the high hydrogen bond density and strength (Fig. 6g). Moreover, the MM/GBSA method of GROMACS was applied to calculate the free binding energies of the 2,3-BPG and PNP complexes to describe the lowest energy conformations from the molecular dynamics simulations. If protein‒ligand interactions are weak, multiple rough low-energy clusters are present in the free energy distribution. Conversely, strong and stable interactions lead to the formation of an almost single and smooth energy cluster in the potential energy distribution^34^. Consistent with the RMSD curve results (Fig. 6g), the free energy distribution diagrams revealed that the 2,3-BPG and PNP complex formed a single minimum energy cluster with high complex stability (Fig. 6h). Therefore, we provide multiple lines of evidence that 2,3-BPG strongly binds to the active site of PNP.

Early studies demonstrated that the active site of PNP includes residues for binding purine, ribose, and phosphate (Pi)^35^. Among all of these residues, Ala116, Ser33, and His86 in PNP were found to interact with its endogenous substrate, phosphate (Pi)^36^. Given that the concentrations of both 2,3-BPG and Pi in RBCs are in the millimolar range^37^ and on the basis of our genetic, molecular modeling, and atomic analyses, we hypothesized that 2,3-BPG inhibits PNP activity by competing with Pi to bind to these phosphate combined residues. To test this hypothesis, we utilized an in vitro competition assay to quantify PNP activity in the presence of free Pi and different concentrations of 2,3-BPG. Using this assay, we observed a decrease in activity when 2,3-BPG was added to the RBC lysate of 4-month-old *eBpgm*^*–/–*^ mice, and increasing the Pi concentration reduced the inhibitory effect (Fig. 6i). This Lineweaver–Burk double reciprocal plot revealed a common intercept on the y axis, indicating competitive inhibition, which is consistent with the active site docking prediction.

Finally, to determine whether 2,3-BPG has an inhibitory effect on PNP activation by competing for binding to these three amino acid residues in the active site of PNP identified by molecular docking and molecular dynamics simulations, we mutated Ser33 and His86 of PNP to alanine and Ala116 to phenylalanine, after which we performed protein expression experiments in HEK293T cells (Fig. 6j). Western blot analyses confirmed the successful expression of the WT and mutated PNP proteins in HEK293T cells (Supplementary Fig. S6b). Consistent with previously published studies^38^, we confirmed that mutation of Ser33, His86 or Ala116 led to a significant decrease in PNP activity in HEK293T lysates (Fig. 6k). To determine which residues are responsible for 2,3-BPG binding to the active sites of PNP and in turn contribute to the inhibitory activity of 2,3-BPG, we added different concentrations of 2,3-BPG to the lysates of HEK293T cells expressing the WT and mutated PNP proteins and subsequently measured PNP activity. The inhibitory effect of 2,3-BPG on PNP activity was maintained in lysates from cells expressing the WT or H86A-mutated PNP protein but completely abolished in lysates from cells expressing the A116F or S33A mutants, indicating that 2,3-BPG exerts an inhibitory effect by interacting with the Ser33 and Ala116 residues of PNP (Fig. 6l, m). Taken together, the results of sophisticated multidisciplinary molecular experiments, including molecular docking, molecular dynamics simulations coupled with in vitro competition functional assays and mutation analyses, revealed that 2,3-BPG acts as an endogenous inhibitor of PNP by competing with free Pi to bind to residues Ser33 and Ala116 in the PNP active site. The gradual decrease in the level of 2,3-BPG in RBCs with age allows for increased PNP activity, thereby promoting inosine catabolism to produce an additional carbon source during aging (Fig. 6n).

## Discussion

**Fig. 7:**
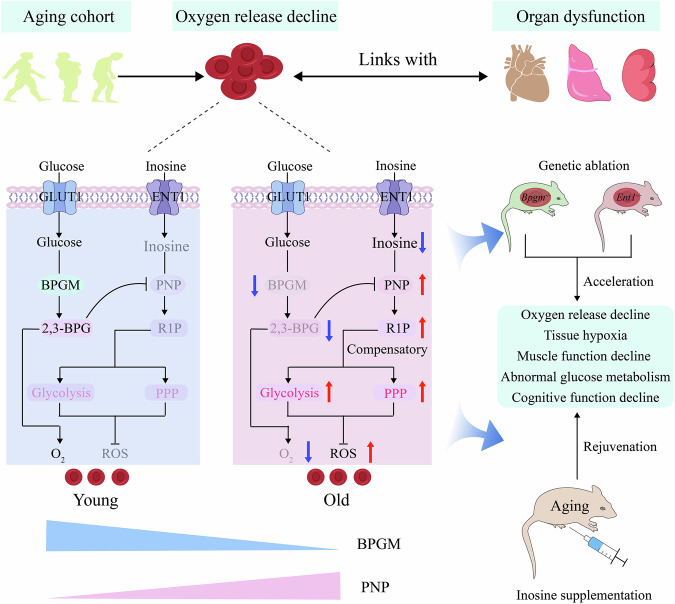
Summary of the work. Our work revealed that decreased BPGM-mediated 2,3-BPG production in erythrocytes, as a newly identified hallmark of aging, contributes to RBC glucose metabolic impairment, RBC functional decline and peripheral tissue hypoxia and dysfunction. We revealed that the ENT1-dependent uptake of inosine and an increase in R1P production from inosine via increased PNP1 activity is a compensatory mechanism that promotes glycolysis and the PPP pathway and triggers oxygen delivery from RBCs to combat age-induced tissue hypoxia and tissue dysfunction. We revealed that the erythroid glycolytic intermediate 2,3-BPG is an endogenous inhibitor of PNP in erythrocytes. As 2,3-BPG levels decrease during aging, its inhibitory effect on PNP diminishes, resulting in increased PNP activity and inosine catabolism as a compensatory mechanism to maintain RBC function and metabolism. Additionally, RBC-specific genetic ablation of BPGM or ENT1 reduces O_2_ release and accelerates aging. Inosine supplementation restored O_2_ release by RBCs, reversed tissue hypoxia, muscle function decline and abnormal glucose metabolism, and mitigated age-related cognitive decline in mice.

Although RBCs are the only cell type that delivers O_2_, the significance of RBCs in aging remains enigmatic. Here, we report that decreased erythroid BPGM-mediated 2,3-BPG production, as a newly identified hallmark of aging, contributes to RBC glucose metabolic impairment, RBC functional decline and peripheral tissue hypoxia and dysfunction with aging (Fig. 7). Moreover, we revealed glucose and purine metabolic crosstalk via the 2,3-BPG-PNP axis during aging and highlight inosine as an alternative fuel to combat impaired glucose metabolic reprogramming and initiate rejuvenation to increase RBC metabolism and oxygen delivery (Fig. 7). Overall, our work adds RBC metabolic and functional decline to a list of hallmarks of aging and positions inosine as an innovative intervention for age-related hypoxia and tissue dysfunction.

O_2_ (known as the “elixir of life”) is essential for maintaining tissue energy metabolism, function and survival^39^. As the most abundant cell type, RBCs are very sensitive to hypoxia, and their role in aging and the underlying molecular mechanisms remain unknown. We report here that the RBC O_2_ release capacity gradually decreases from an early age, with no significant differences between sexes; these findings are consistent with those of our previous study^40^. Similar to earlier findings, the elderly group in our cohort displayed aging-related clinical outcomes, including higher levels of inflammation^41^, liver and kidney dysfunction^42^, and impaired glucose‒lipid metabolism^43^, than young individuals did. Additionally, we revealed that decreased O_2_ release capacity is strongly linked to age-related metabolic impairment and tissue dysfunction. Our findings highlight the novel and compelling concept that an age-dependent decline in RBC function is a previously unrecognized hallmark of aging that is strongly linked to metabolic impairment and age-related functional decline in multiple tissues.

Mature RBCs lack nuclei and mitochondria^44^ and act as both a deliverer and sensor of O_2_^45^, which is vital for maintaining the normal function and survival of every cell within the body. Owing to the large amount of O_2_ carried by RBCs, they contain a well-defined system to defend against oxidative stress^46^. Thus, RBCs rely on sophisticated glucose metabolic reprogramming, including glycolysis, RLS and the PPP, to effectively deliver O_2_ and maintain their anti-oxidative stress capacity^25^. Notably, one of the most well-known allosteric modulators is 2,3-BPG, a byproduct of glycolysis known for regulating hemoglobin O_2_ binding affinity, which is generated primarily by BPGM, a key enzyme in the RLS that is highly enriched in RBCs^45,47^. Consistent with our comprehensive metabolomics findings, early studies reported that the RBC 2,3-BPG concentration in humans decreases with age^48^. In contrast, we revealed that glycolysis, the PPP and purine catabolism are significantly activated in RBCs during aging. These findings are strongly supported by early studies that revealed that the activity of hexokinase and pyruvate kinase, which are involved in glycolysis, did not significantly change in RBCs during aging but is significantly increased in patients with Alzheimer’s disease^47^. Here, we demonstrated that BPGM activity decreases with increasing age in humans. Functionally, we provide genetic evidence that erythroid-specific ablation of BPGM in mice decreases 2,3-BPG production and O_2_ delivery and accelerates age-related functional decline. Thus, both human and mouse genetic studies have added a significant new chapter to our understanding of erythrocyte BPGM-mediated 2,3-BPG production during aging. Furthermore, the PPP is critical for RBCs to produce sufficient amounts of NADPH and thus maintain an abundance of GSH, which acts as a ROS scavenger to defend against cellular oxidative stress^48^. Consistent with the findings of early studies^49^, we observed elevated ROS levels along with increased PPP activity and GSH levels in our aging study. Our metabolomics results revealed that the reduction in 2,3-BPG production caused by decreased BPGM activity is key to the decrease in oxygen release by RBCs, whereas the activation of the PPP is the compensatory metabolic basis for resistance to the increased levels of ROS. Further genetic studies in mice provided proof-of-principle evidence that decreased erythrocyte BPGM-mediated 2,3-BPG production is an essential component underlying aging-induced erythrocyte glucose metabolic impairment, RBC functional decline and peripheral tissue hypoxia and dysfunction with aging. This work highlights the novel and compelling concept that erythroid BPGM-mediated glucose metabolic impairment is a central mechanism and “previously overlooked” hallmark of aging underlying reduced RBC function and thereby driving age-dependent tissue hypoxia and dysfunction.

Glucose is the major fuel that maintains RBC bioenergetics, and our findings, including those from both the human and mouse genetic studies, show that inosine, which is taken up by an ENT1-dependent mechanism, is an additional fuel for RBCs owing to increased PNP-driven inosine catabolism, compensating for the impaired glucose metabolism in these cells during aging. Our results show that in RBCs from older adults, inosine-derived ribose is directed toward glycolysis, PPP and RLS to counteract the age-dependent decline in RBC function. The concentration of inosine in RBCs is in equilibrium with that of extracellular inosine via the action of ENT1. Early studies indicated that ENT1 is highly expressed in RBCs^31^ and that ENT1-mediated adenosine uptake plays an essential role in increasing O_2_ delivery by inducing A2B-AMPK-dependent BPGM activation in the contexts of chronic kidney disease^29^ and fetal growth restriction^50^. Notably, earlier studies revealed that inosine or adenosine helps preserve ATP and 2,3-BPG levels in stored RBCs^51^, suggesting that these compounds may serve as preservatives for the storage of both blood and other organs^52^. Recent studies have demonstrated that inosine increases fat burning or provides energy in adipocytes, cancer cells and T cells through either adenosine receptors or the transporter ENT1^53–55^. In contrast to the increased PNP activity-mediated inosine catabolism, we detected no changes in ADA activity during human aging. These findings led to our discovery that adenosine-induced erythrocyte O_2_ delivery and anti-oxidative stress capacity are independent of ADA and PNP and further confirmed an early report that adenosine functions primarily via its surface receptor, ADORA2B, to improve RBC function^30^. Our findings are strongly supported by those of previous studies showing that unlike inosine, adenosine does not serve as an alternative carbon source to enhance glycolysis and the TCA cycle in T cells^55^. However, inosine-mediated metabolic reprogramming in erythrocytes and its specific function and underlying mechanisms during aging remained unresolved prior to our current studies. Here, we provide initial in vitro and in vivo evidence that ENT1-PNP-facilitated inosine uptake and metabolism are essential for directly inducing O_2_ offloading and anti-ROS activity. We also provide in vivo evidence that specific genetic ablation of ENT1 in mouse RBCs led to a decrease in O_2_ release from RBCs and accelerated aging. There is currently no approach to promote oxygen delivery to combat aging. Extending upon the results from genetic studies, we provide both in vitro and in vivo preclinical evidence that inosine can directly increase O_2_ delivery from RBCs, mitigate tissue hypoxia and slow aging-related functional decline. Therefore, our findings support the novel concept that glucose is not the only fuel for RBCs and that inosine-derived R1P metabolized by PNP owing to its increased activity is an additional fuel that can compensate for improper glucose metabolism to combat aging-related tissue hypoxia, organ damage and dysfunction. These findings are extremely significant and provide a foundation for future translational studies on rejuvenation and even all types of physiological and pathological hypoxia.

Emerging studies have reported that 2,3-BPG is involved in the regulation of various protein functions in addition to directly binding to Hb, particularly in the areas of O_2_ transport and metabolic regulation. For example, recent research revealed that 2,3-BPG is involved in phosphorylating a histidine residue in phosphoglycerate mutase 1 (PGAM1), which catalyzes the conversion of 3-PG to 2-PG, increasing its activity and thus regulating energy metabolism^56^. Additionally, hypoxanthine, IMP, and inosine are components of the oxypurine cycle in RBCs. Early studies reported that the oxypurine cycle is significantly correlated with 2,3-BPG levels^57^. In addition, previous research revealed that 5’-nucleotidase might be activated by 2,3-BPG, which promotes IMP phosphorylation^58^. Furthermore, early studies have shown that 2,3-BPG can inhibit AMP deaminase activity in various animal tissues^59^. These studies suggest that 2,3-BPG is involved in regulating purine metabolism. However, the role of 2,3-BPG in the regulation of PNP activity remained unknown prior to the current study. Our human metabolomics results unexpectedly revealed that R5P or R1P and elevated PNP enzyme activity are significantly negatively correlated with the level of 2,3-BPG. Our human metabolomics findings are strongly supported by early studies showing that during storage, BPGM activity and 2,3-BPG levels decrease, while PNP enzyme activity increases^60^. Here, we provide proof-of-principle genetic evidence that PNP enzyme activity is significantly increased in mice with erythroid-specific BPGM mutation. The active site of PNP can be divided into three binding regions depending on the moiety it accommodates: the purine binding and sugar binding regions accommodate substrate moieties, while the phosphate binding region accommodates inorganic phosphate. Crystal structure studies have revealed that the residues Ser33, His86, and Ala116 of PNP, especially Ser33 and His86, interact with Pi through direct hydrogen bonding^38,61^. Early studies demonstrated that acyclovir diphosphate can bind directly to the phosphate binding site of PNP and compete with Pi, thereby reducing the reaction rate^62^. The phosphate-containing drug developed by Zlatko Janeba also binds to these sites, forming hydrogen bonds and increasing the efficacy of PNP inhibitor drugs^63^. Intriguingly, our molecular docking and molecular dynamic simulations revealed that 2,3-BPG has the potential for strong binding to the Pi binding site and that the two phosphate groups of 2,3-BPG provide the possibility for phosphate competition. Our phosphate competition experiments confirmed the competitive inhibitory effect of 2,3-BPG on PNP activation. Moreover, PNP site-directed mutagenesis provided functional evidence that 2,3-BPG inhibits PNP enzyme activity primarily by binding to Ser33 and Ala116. Overall, we determined that 2,3-BPG acts as an endogenous inhibitory modulator of PNP activity by competitively binding to the Pi site in mature RBCs. Thus, the 2,3-BPG-induced decrease in PNP activity is a compensatory response to increase inosine catabolism and generate additional carbon sources to combat glucose metabolic impairment during aging. These findings are extremely innovative and add a significant new knowledge regarding RBC biology and metabolism during aging.

In conclusion, in both human and murine studies, we revealed that the BPGM-dependent decline in RBC bioenergetics is a significant and novel hallmark of aging and plays a critical role in driving age-induced tissue hypoxia and dysfunction. At the cellular and metabolic levels, we further revealed that the ENT1-dependent uptake of inosine and increase in PNP-mediated R1P production from inosine is an alternative fuel to counteract glucose metabolism impairment, induce oxygen delivery by RBCs and combat age-induced tissue hypoxia and tissue dysfunction. At the molecular level, we revealed that the erythroid glycolytic intermediate 2,3-BPG is an endogenous inhibitory modulator of PNP, a key enzyme of purine metabolism. Thus, the age-dependent decrease in 2,3-BPG content increases erythroid PNP activity and inosine catabolism to produce an alternative fuel to overcome impaired glucose metabolism and promote oxygen delivery as novel compensatory glucose-mediated purine metabolism machinery. Overall, our study significantly advances our understanding of aging from a completely new angle, elucidating the dysfunction of RBCs during aging from a metabolic perspective and proposing a novel concept in which tissue damage during aging is a result of the impairment of oxygen release from erythrocytes. By repositioning RBCs as active regulators of healthy aging, we open new avenues for rejuvenation.

## Materials and methods

### Volunteer recruitment and data collection

The Hunan Aging Cohort Study was established in Hunan Province, China, and was conducted with the approval of the Research Ethics Committee of Xiangya Hospital of Central South University with informed consent from volunteers (202310207). The collection of biological samples and data in this study was performed in accordance with the guidance of the Human Genetic Resource Administration, Ministry of Science and Technology of the People’s Republic of China.

The blood samples and clinical information from 301 recruited individuals are shown in Supplementary Table S1. All volunteers were considered qualified and included in this study on the basis of the following criteria: (1) healthy adults over 18 years of age; (2) no severe diseases (such as cancer, cardiovascular disease, diabetes mellitus, autoimmune/inflammatory, or severe gynecological disease); (3) no persistent drug or alcohol abuse; (4) no other clinical trial participation within the past three months; (5) no use of hormone medication (past six months), anti-platelet drugs, or cholinesterase inhibitors for the treatment of Alzheimer’s disease; and (6) no extreme vigorous exercise, acute injury or other abnormal behaviors for one month before sample collection. For young females with regular menstrual cycles, blood was collected one week before menstruation.

### Animals

All animal protocols were performed in accordance with the National Institutes of Health guidelines and approved by the experimental animal ethics committee of Central South University, Changsha, Hunan Province, China (CSU-2023-0336). All experimental animals were housed under controlled conditions at a temperature of 22 °C with 50% humidity and lights programmed to be on from 7 am to 7 pm. Food and water were available ad libitum. Mice with erythrocyte-specific deletion of BPGM (e*Bpgm*^–/–^ mice) were generated by crossing mice that were homozygous for a floxed *Bpgm* allele with *EpoR-Cre*^+^ mice. Mice with erythrocyte-specific deletion of ENT1 (e*Ent1*^–/–^ mice) were generated by crossing mice homozygous for a floxed *Ent1* allele with *EpoR-Cre*^+^ mice. Four-month-old and 15- and 16-month-old C57BL/6 WT mice were purchased from Aniphe Biolaboratory, Inc. C57BL/6 WT mice were bred in a pathogen-free, temperature- and humidity-controlled facility on a 12-hour light and dark cycle. Sick animals were excluded from the study. Mice were labeled with ear tags, and all the researchers were blinded to the experimental procedures.

### Human biological sample pretreatment and storage

All blood samples were collected after an overnight fast to avoid dietary influence. Each 10 mL venous whole blood sample was collected from individuals by a professional nurse. One 5 mL blood sample was collected in an EDTA anticoagulant tube, while another 5 mL sample was collected in a standard tube. After collection, the tubes were immediately sent to the laboratory and stored at 4 °C, after which the biochemical tests were performed. The blood in the EDTA tube was centrifuged at 4 °C (500× *g*, 10 min) to separate the plasma and blood cells. Plasma and RBCs were further centrifuged at 4 °C (4000 rpm, 10 min) to obtain the plasma, platelets, and RBCs. All blood samples were stored at –80 °C for long-term preservation.

### Measurement of P50 and ROS levels of RBCs

To measure the P50, 20 μL of whole blood was added to 3 mL of Hemox buffer (TCS Scientific Corporation) with 5 μL of anti-foaming reagent (TCS Scientific Corporation) and 5 μL of 22% BSA in PBS. The mixture was then analyzed with a Hemox analyzer (TCS Scientific Corporation) for data measurement at 37 °C and subsequent construction of the oxygen equilibrium curve. The Hemox analyzer was used to determine the P50 from the oxygen equilibrium curve at 37 °C. The ROS level was measured using a ROS Assay Kit (BL714A, Biosharp) according to the manufacturer’s protocol. Briefly, 1 μL of RBC pellet was incubated with a ROS fluorescence probe (H2DCFDA, 2 μM) for 45 min at 37 °C in the dark. The samples were then washed 3 times with PBS and resuspended in 200 μL of PBS. ROS levels were determined by measuring the fluorescence intensity with a microplate reader (Synergy HTX Multi-Mode Reader, BioTek).

### Intraperitoneal administration of inosine or vehicle to mice

The effect of inosine was investigated in aged 16-month-old C57BL/6 male mice. Mice were injected intraperitoneally with vehicle (90% saline) or inosine (20 mg/kg body weight) daily for 1 month and culled at the age of 17 months for terminal assays. The dosage of inosine was determined according to published articles related to inosine treatment^64^. For consistency, rigor, and reproducibility, mice were injected at the same time each day (10:00 am), each physiological assay was performed at the same time of the day (indicated separately for each assay), and all animals were fed the same normal chow.

### Health analysis

The physiological functions and health of various organs in 17-month-old mice were assessed after inosine supplementation and included the following: body weight, blood pressure, rotarod and grip strength tests to indicate neuromuscular strength, and the NOR and Barnes maze tests to examine behavior (functional tests are described below). Blood collected from 17-month-old mice was subjected to biochemical testing and P50 measurement.

### Blood pressure measurement

Mouse blood pressure was measured using a noninvasive BP system (CODA High Throughput System with 8 Activated Channels; Kent Scientific; Torrington, CT) as previously described^19^. Briefly, mice were placed on a temperature-controlled platform such that the tail temperature was between 32 °C and 35 °C, and blood pressure was monitored using a volume pressure recording sensor. Training was conducted once a day for 3 days before minipump implantation. For each mouse blood pressure measurement, the first 5 cycles were considered the adaptation period, followed by 20 additional cycles of recorded blood pressure measurements. The average of these 20 cycles was used for blood pressure analysis.

### Rotarod test

Motor coordination and balance were tested using a rotarod apparatus as described previously between 10:00 am and 12:00 noon^65^. The apparatus consists of a plastic rod (diameter of 6 cm) and five equal sections separated by six disks (each with a diameter of 25 cm), which allows five mice to be tested simultaneously. Mice were trained on the rotarod apparatus for 3 consecutive days before the test day. On the test day, mice were acclimated to the environment for 30 minutes. The time at which the mice fell off the rod (rotating at a speed of 10 or 30 rpm) was recorded. There was a 5 min break between each speed test. The mean latency time of falling off the apparatus was taken as the performance value.

### Grip strength measurement

The grip strength test was used to measure the maximal muscle strength of the forelimbs. The test was based on assessing a mouse grasping a grid with sensors, as previously described^65^.

### NOR test

The NOR test was performed as described previously^66^. The mice were placed in an open-top, transparent acrylic resin box with bedding that was divided into 4 quadrants (Q1, Q2, Q3, and Q4). Each mouse was initially placed in the empty chamber and allowed to freely explore the arena without objects for 10 minutes to adapt to the test environment. Next, the mice started the training procedure, in which each mouse was placed in the arena with 2 identical objects (Q2Block+Q3Block) for 5 minutes each day for a total of 3 days. On Day 3, the final trials were performed by placing the mice in the arena and exposing them to one of the previous objects and a novel object (Q2Block+Q3Cylinder) for 5 minutes. The recognition times and motion trajectories associated with the novel object were recorded by TopScan 2.0 software (CleverSys) and used for subsequent analysis.

### Barnes-maze test

The Barnes maze test was performed as described previously^66^. A circular platform with 40 holes and surrounded by visual clues was set as the arena. One stationary escape box was set on one hole that was not changed throughout the experiment for mice the to navigate to, with negative stimuli (buzzer and light) provided as clues to exit. On Day 1, each mouse was placed in the area and guided by the researcher to the escape box without negative stimuli for familiarization. Next, 2 rounds of adaptation were performed, which were the same as the familiarization procedure but included the negative stimuli. Two rounds of acquisition trials were completed by placing the mice in the arena and allowing them to explore freely to find the exit for no more than 3 minutes. Four acquisition trials were completed on Days 2 to 5. Days 1 to 4 of the trials were termed the training period. Then, the next day after training, the short-term memory was assessed, and the long-term memory was assessed one month or one week after treatment. Primary latency to reach the escape box was recorded by the TopScan system and used to assess spatial learning and memory.

### Glucose tolerance tests

For the glucose tolerance tests, glucose (2 g/kg BW) was injected intraperitoneally (i.p.) after an overnight (16 h) fast, and blood glucose was monitored using blood glucose strips and an Accu-Check glucometer (Roche) at indicated times as described previously^67^. Afterward, the area under the curve was calculated using the R software.

### Detection of tissue hypoxia

The level of hypoxia in heart, liver, kidney, muscle, and brain tissue was assessed using a Hypoxyprobe kit (Hypoxyprobe Omni Kit, Hypoxyprobe, Inc.) according to previously described methods^29^. Briefly, mice were intraperitoneally injected with pimonidazole (50 mg/kg body weight) 30 min before anesthesia. Transcardiac perfusion was performed before organ collection, after which the tissues were fixed in 10% buffered formalin and embedded in paraffin to prepare 4-micron sections. The sections were deparaffinized by a series of immersions in xylene and rehydrated in a graded series of alcohol and distilled water. Antigen retrieval was performed by boiling the sections in sodium citrate buffer (pH 6.0) at 95 °C for 30 min. After blocking with 3% BSA in PBS at room temperature for 1 h, the sections were incubated overnight with rabbit anti-pimonidazole antibody (PAb2627AP, 1:100 in 0.5% BSA) in a humid environment at 4 °C. Following primary antibody incubation, incubation was performed with the Alexa Fluor 594-labeled goat anti-rabbit secondary antibody (A-11037; Thermo Fisher; 1:1000 in 3% BSA in PBS). Negative control samples were incubated with the secondary antibody only. The sections were mounted with ProLong Gold antifade reagent containing DAPI (P36935; Invitrogen). The images were imported into ImageJ, and the percentage of the area with positive staining for a certain antibody relative to the total structural area was calculated under the same threshold settings. Afterward, the fluorescence signal intensity was quantified.

### Histological analysis

Mouse tissues (heart, liver, kidney, muscle, and brain) were fixed in 10% buffered formalin and sent to Servicebio Biotechnology Co., Ltd. (Hubei, China) to be prepared into 4-micron sections and subsequent hematoxylin‒eosin staining or Nissl staining. The slides were examined under a light microscope to detect tissue damage. After being embedded in Tissue-Tek optimal temperature compound (Sakura Finetek, Torrance, CA, USA), 10 μm sections of the brains were prepared on a cryostat, mounted on slides, and stored at –20 °C. Coronal brain sections with similar anatomical locations near the bregma (−2 mm) were selected according to the mouse brain atlas. The frozen sections were washed with PBS, blocked, and permeabilized with 0.3% Triton X-100 (Sigma, St. Louis, MO, USA) and 10% BSA (Sigma) in PBS at room temperature for 2 h. Sections were incubated with primary antibodies diluted in blocking buffer (0.3% Triton X-100 and 10% BSA in PBS) overnight at 4 °C. After being washed 3 times with PBS for 5 minutes each, the sections were incubated with secondary antibodies diluted in blocking buffer for 1 h at room temperature. After being washed 3 times (5 min each), the sections were mounted with ProLong Gold Antifade Mountant with DAPI (P36935; Invitrogen). Primary antibodies used were anti-GFAP (Invitrogen, Cat# Pa1-10019; 1:500; Waltham, MA, USA) and anti-Iba1 (Wako, Cat# 019-19741; 1:500; Osaka, Japan). Image acquisition was performed using a Zeiss LSM 880 confocal microscope, and the images were processed using Zen Blue Edition Lite 3.0 software (Carl Zeiss, Oberkochen, Germany) to generate maximum intensity projections of each image using a 20× objective lens through the tiling and positioning module and Z-stacks. Afterward, the Iba1 and GFAP fluorescence signal intensities were quantified using ImageJ software. The images were imported into ImageJ, and the percentage of the area with positive staining for a certain antibody relative to the total structural area was calculated under the same threshold settings.

### Western blotting

Western blot analysis was performed as previously described^29^. Briefly, 25 mg of tissue was ultrasonically lysed in cold water supplemented with 1× protease inhibitor cocktail (Roche, Cat# 04906837001) at a ratio of 1:10, after which 10× PBS was added for equilibration. Protein concentration was measured using a BCA protein assay kit (ThermoFisher Scientific). The samples were loaded onto 10% SDS‒PAGE gels for Western blot analysis. Total protein was incubated with primary antibodies against BPGM (Proteintech, #17173-1-AP, 1:2000) or β-actin (Proteintech, #66009-1-Ig, 1:3000), followed by incubation with secondary antibodies.

### Metabolomics analysis

The RBC samples were thawed at 4 °C, and 20 μL aliquots were mixed with 200 μL of a mixture of cold methanol/acetonitrile (methanol:acetonitrile:water 5:3:2 v/v/v) to remove the protein. The mixed samples were then vortexed continuously for 30 min at 4 °C and then centrifuged at 18,000× *g* for 10 min at 4 °C. Supernatants were injected onto the ultrahigh-pressure liquid chromatography‒mass spectrometry (UHPLC‒MS) system composed of a Vanquish UHPLC coupled to a Q Exactive mass spectrometer (Thermo Fisher Scientific, Bremen, Germany) to obtain RBC metabolic fingerprints using a 5-minute gradient as previously described^29^. Briefly, the metabolites were separated with a Kinetex C18 column (Waters, 150×2.1 mm, 1.7 μm; Phenomenex, 00F-4475-AN). The injection volume was 5 μL, and the flow rate was 0.4 mL/min. The column temperature was maintained at 45 °C, and the autosampler temperature was set at 4 °C. For detection in positive ion mode, the mobile phase consisted of 0.1% formic acid in water (A) and 0.1% formic acid in acetonitrile (B) with gradient elution as follows: 0–0.5 min at 5% B; 0.5–1.1 min from 5–95% B; 1.1–2.75 min at 95% B; 2.75–3 min from 95–5% B; and 3–5 min at 5% B. In negative ion mode, mobile phases were composed of 1 mM ammonium acetate in 95% water (A, 5% acetonitrile) and 1 mM ammonium acetate in 95% acetonitrile (B, 5% water) with gradient elution as follows: 0–0.5 min at 0% B; 0.5–1.1 min from 0–100% B; 1.1–2.75 min at 100% B; 2.75–3 min from 100–0% B; and 3–5 min at 0% B. The mass spectrometer was operated in full MS mode at a resolution of 70,000, the scan range was 65–900 m/z, the maximum injection time was 200 ms, 2 microscans were performed, automatic gain control was set to 33106 ions, the source voltage was 4.0 kV (for both positive and negative ion modes), the capillary temperature was 320 °C, and the sheath gas (45), auxiliary gas (15), and sweep gas (0) were all nitrogen. Samples were randomly injected in positive and negative ion modes independently. The QC sample was injected six times prior to sample analysis and then injected at regular intervals (every ten samples) thereafter to monitor the stability of the system. The QC samples were prepared as composite samples and contained 5 μL of each sample.

The raw MS data were first converted to mzXML files using RawConverter (Scripps Research Institute) and then subjected to peak picking (mz diff = 10 ppm) and grouping (bw = 5, mz window = 0.025, minfrac = 0.2) with EI-Maven software (version 0.12) on the basis of the endogenous metabolite database HMDB. The isotopic abundance ratios of metabolites were calculated using EI-Maven software. Metabolite identification was conducted by matching the metabolite isotopic relative abundance ratio values and comparing the accurate m/z values with MS/MS spectra with an in-house database.

### In vitro RBC intervention experiments

RBCs were isolated from the blood of young (30 years) and elderly (71 years) humans, WT mice, and RBC-specific knockout mice using sodium heparin. For in vitro glucose flux experiments in which the RBCs were treated with inosine, erythrocytes were isolated from the blood of young volunteers using sodium heparin as an anticoagulant. The erythrocytes were subsequently washed 3 times with F-10 Nutrient Mix (Invitrogen, #0905074EF), suspended to 10% hematocrit, and then transferred to 1.5 mL tubes in triplicate. For metabolite treatment, different concentrations of inosine (5 μM, 50 μM, 100 μM, and 500 μM) were added to the erythrocytes, which were then incubated for 1 h, after which the P50 was measured to determine the optimal concentration. Then, the erythrocytes were incubated with 100 μM inosine ± 5 μM dipyridamole (an ENT1 inhibitor), 100 μM inosine ± 5 μM forodesine (a PNP inhibitor), 100 μM adenosine, 100 μM adenosine ± 5 μM forodesine, 100 μM adenosine ± 5 μM 2’-deoxycoformycin (an ADA inhibitor), or adenosine ± 20 μM PSB1115 (an ADORA2B inhibitor) for 1 h at 37 °C. Finally, the erythrocytes were washed three times in cold PBS, after which the P50 and ROS concentrations were measured.

### ^13^C_5_-Inosine tracing experiments with cultured RBCs

Erythrocytes were isolated from the blood of young (30 years) and elderly (71 years) humans using sodium heparin as an anticoagulant for inosine metabolic flux experiments. The erythrocytes were washed 3 times with F-10 Nutrient Mix (Invitrogen), suspended to 10% hematocrit, and then transferred to 1.5 mL tubes in triplicate. For certain experiments, erythrocytes (20 μL) were cultured in the presence of 100 μM [^13^C_5-_ribose]-inosine (MedChemExpress) for 0.5 h, 1 h, or 2 h at 37 °C (3 samples at each time point). Next, the erythrocytes were washed three times with ice-cold PBS and subjected to metabolic quenching in cold acetonitrile. Finally, erythrocytes and supernatants were collected and lysed as described above and analyzed by the Vanquish UHPLC coupled to a Q Exactive MS (Thermo Fisher Scientific, Bremen, Germany). Metabolite identification and isotopologue distributions were performed with EI-Maven software (Princeton, NJ) and the R package AccuCor^68^.

### PNP activity assay

PNP enzyme activity was determined according to previous methods^69^. Briefly, RBCs or 293 T cells were diluted (1:1) in PBS containing 0.3% Triton X-100. PNP activity was determined spectrophotometrically by adding 1 μL of the cell lysate to 200 μL of reaction buffer containing 50 mM KH_2_PO_4_ (pH 7.5), 1 mM inosine, and 10 mU xanthine oxidases (Sigma). Reaction progress was monitored at 293 nm for 30 min at 30 °C. After the reaction was complete, RBC hemoglobin concentration was determined by measuring the absorbance at 414 nm, and the PNP activity was normalized to the hemoglobin concentration. The total protein levels in the cell lysates were determined using a BCA protein assay kit, and the PNP activity of the 293 T cells was normalized to the total protein concentration.

### ADA activity assay

ADA activity was determined by measuring inosine production by coupling the ADA reaction with purine nucleoside phosphorylase and xanthine oxidase reactions^70^. ADA activity was assayed under saturated substrate conditions at 37 °C in a reaction mixture containing 2 mM adenosine, 10 mU xanthine oxidases, 10 mU purine nucleoside phosphorylase, and 50 mM sodium phosphate (pH 7.4), in a total volume of 200 μL, of which 1 μL was the cell lysate. The increase in absorbance at 293 nm resulting from the deamination of adenosine to yield inosine and urate was continuously monitored with a spectrophotometer, and the rate of inosine production was calculated by linear regression.

### 2,3-BPG competition PNP enzyme assays

A 0.1 mg/mL solution of purine nucleoside phosphorylase (PNP; 1ku; HARVEY BIO) in 100 mM Tris/HCl (pH = 7.5) was prepared. The enzymatic activity assay was performed, and the enzyme unit was defined as previously described^71^. The enzymes were assayed spectrophotometrically at 25 °C. The standard reaction mixture contained 1 mM inosine in sodium phosphate buffer (different concentrations, pH 7.5) and xanthine oxidase (0.05 U/mL) in a final volume of 0.2 mL. The phosphorolysis of inosine was similarly assessed by measuring the absorbance at 293 nM in the presence of xanthine oxidase (0.05 U/mL) and different concentrations of 2,3-BPG. The reaction rate, as determined by the measured increase in A293 due to the formation of uric acid, was proportional to the enzyme concentration. One enzyme unit is defined as the amount of enzyme that catalyzes the phosphorolysis of 1 μmol of inosine/min, corresponding to an increase in A293 under the conditions described. Specific activity is expressed as units/mg of protein or Hb.

### BPG mutase activity assay

Ten microliters of erythrocytes were lysed in Tris-HCl buffer (pH 7.4) containing 0.5% Triton X-100 and protease inhibitors (Roche, Cat# 04906837001). Then, 5 μL of erythrocyte lysate was incubated at room temperature for 30 min in a 100 μL reaction mixture containing 100 mM triethanolamine (pH 7.6), 1 mM magnesium sulfate, 4 mM adenosine triphosphate (ATP), 3 mM 3-phosphoglycerate, and 10 units of phosphoglycerate kinase. Afterward, 5 μL of 11.63 M perchloric acid was added to stop the reaction, and the sample was subsequently centrifuged at 10,000× *g* for 5 min at 4 °C. The supernatant (8 μL) was mixed with 10 μL of 2.5 M K_2_CO_3_, vortexed, and centrifuged at 10,000× *g* for 5 min at 4 °C. Finally, 10 μL of the supernatant was used for 2,3-BPG quantification using a commercially available kit as previously described^72^.

### Molecular docking prediction of the 2,3-BPG binding site in PNP

Molecular docking was used to predict the binding affinity between 2,3-BPG and PNP. The three-dimensional PNP structure was obtained from the UniProt database, and the two-dimensional structure of 2,3-BPG was obtained from the PubChem database (https://pubchem.ncbi.nlm.nih.gov/) and converted into a three-dimensional structure using Chem3D software. All the small molecule was converted into Pdb format using Open Babel for energy minimization in a pH 7.4 environment, followed by hydrogenation with PyMOL (https://www.pymol.org/), and converted into PDBQT format. Afterward, the affinity between 2,3-BPG and PNP was calculated using AutoDock Vina (https://vina.scripps.edu/)^73^. An affinity ≤ –5.0 kcal/mol indicated a strong interaction. PyMOL software was used to visualize the results. The CavityPlus online tool (https://www.pkumdl.cn/cavityplus) was used to predict the active pockets of the PNP protein, and the best active pocket was determined on the basis of DrugScore and Druggability rankings^74^.

### Molecular dynamics simulations

Molecular dynamics simulations of the 2,3-BPG-PNP complexes were performed using GROMACS 2022.4 software^75^. Amber Tools was used to add GAFF force field to the small molecule, whereas Gaussian 16 W is used to add hydrogen atoms to the small molecule and calculate the RESP potential^76^. Potential data was added to the topology file of the molecular dynamics system. The simulations were carried out at a static temperature of 300 K and atmospheric pressure (1 bar). Amber99sb-ildn was used as a force field, water molecules were used as solvent (TIP3P water model), and the total charge of the system was neutralized by adding an appropriate number of Na^+^. The steepest descent method was applied to minimize the energy, and then equilibration was performed with the isothermal isovolumic (NVT) ensemble and isothermal isobaric ensemble (NPT) ensemble for 100,000 steps, with a coupling constant of 0.1 ps for a total duration of 100 ps. Finally, free molecular dynamics simulations were performed. The process consisted of 5,000,000 steps, the step length was 2 fs, and the total duration was 100 ns. After the simulation was completed, the built-in tool of the software was used to analyze the trajectory, and the RMSD, root-mean-square fluctuation (RMSF), and protein radius of gyration of each amino acid trajectory were calculated and combined with the free energy (MMGBSA), free energy topography, and other data.

### Correlation analysis

Clinical phenotype and metabolomics data correlation analyses were performed using the pcor.test function of the *ggcor* R package (version 1.1) via the Pearson correlation method to determine aging-related characteristics and the relationship between erythrocytes and organ function.

### Calculation of age-related change score

The age-related change score measures the change tendency of group features with age and is calculated on the basis of different functional signatures. The GSVA algorithm^77^ from the GSVA R package was applied to evaluate the age-related change score of each sample.

### Statistical analysis

All the data were analyzed using the R language (version 4.4.1). Each dataset was normalized to the sum of the spectral integrals and subjected to Pareto scaling. Statistical significance was determined using an unpaired two-tailed Student’s *t* test or one-way ANOVA with Dunnett’s post hoc multiple comparisons analysis for comparisons of three or more groups. Pathway enrichment analysis of the metabolites was conducted using the *clusterProfiler* R package^78^. The visualization, correlation network, and pathway enrichment results were performed using the *igraph* packages and *ggplot2* R packages, respectively.

## Supplementary information

Supplemental Material

## Data Availability

Differential metabolite datasets and tracing datasets underlying individual display items in the manuscript are included in Supplementary Data S1, S2. This paper does not report the original code.
