# Supplementary material for: Inosine promotes erythrocyte metabolic reprogramming and restores oxygen release for rejuvenation via 2,3-BPG-PNP axis
Source: Cell Discov. 2026 Mar 17;12:19. doi: 10.1038/s41421-026-00877-6 (PMC12996605; doi:10.1038/s41421-026-00877-6)
Supplement: Supplementary file 1 — Supplemental Material [file 41421_2026_877_MOESM1_ESM.pdf]

Supplementary Information

Supplementary Table S1. The clinical characteristics of aging populations (median-IQR)

Variable	Female (n=133)	Male (n=168)
Age	55.03 (39,71)	54.98 (39,70.75)
Waistline	75.72 (69,83)	84.72 (79,91)
Hipline	88.5 (84,93)	92.46 (88,96)
BMI	22.56 (20.75,24.06)	24.36 (22.22,26.34)
SBP (mmHg)	122.92 (106.75,136.25)	127.1 (112,139.5)
DBP (mmHg)	75.42 (68,82.5)	79.14 (73,86)
MAP (mmHg)	88.31 (80.67,99.42)	92.73 (86.33,102.17)
P50 (mmHg)	25.18 (23.81,26.89)	24.87 (23.69,25.98)
HCT (%)	39.8 (38.2,41.8)	44.65 (43.1,46.5)
RBC (10^{12}/L)	4.38 (4.22,4.55)	4.86 (4.58,5.07)
Hb (g/L)	133.05 (127,139)	149.32 (144,155.75)
MCV (fL)	90.95 (88.7,93.6)	92.11 (89.7,94.77)
MCH (pg)	30.35 (29.6,31.3)	30.83 (30,31.5)
MCHC (g/L)	333.68 (330,338)	334.75 (330.15,339)
RDW (%)	13.72 (13.2,14.3)	13.65 (13.2,14.1)
WBC (10^9/L)	5.9 (4.9,6.9)	6.22 (5.4,6.9)
Plt (10^9/L)	236.64 (190,263)	215.85 (179,247.75)
N (10^9/L)	3.44 (2.7,4)	3.5 (2.8,4)
L (10^9/L)	1.9 (1.5,2.3)	2.01 (1.5,2.3)
Eso (10^9/L)	0.11 (0,0.1)	0.19 (0.1,0.2)
Baso (10^9/L)	0.01 (0,0)	0.02 (0,0.02)
Mono (10^9/L)	0.42 (0.3,0.5)	0.48 (0.4,0.5)
N%	57.7 (53,62.7)	56.02 (50.45,61.48)
L%	32.7 (27.7,37.4)	32.59 (26.63,37.08)
Baso%	0.59 (0.4,0.7)	0.57 (0.4,0.7)
Eso%	1.89 (0.9,2.4)	3.05 (1.4,3.77)
Mono%	7.12 (6,8.1)	7.77 (6.82,8.6)
PCT (ng/mL)	0.21 (0.17,0.23)	0.19 (0.16,0.21)
MPV (fL)	8.87 (8.24,9.4)	8.91 (8.2,9.5)
PDW (%)	16.64 (16.2,16.9)	16.73 (16.3,17.1)
TP (g/L)	74.17 (72,76.3)	74.19 (71.23,76.77)
ALB (g/L)	44.91 (43.1,46.5)	45.06 (43.42,46.68)
GLOB (g/L)	29.26 (27.4,30.9)	29.13 (27.02,31.2)
AGratio	1.55 (1.4,1.7)	1.57 (1.4,1.7)
TBIL (μmol/L)	13.88 (10.3,15.7)	15.28 (11.1,17.5)

DBIL (μmol/L)	3.83 (2.8,4.6)	4.25 (3.1,5)
TBA (μmol/L)	3.86 (1.8,5.1)	4.79 (2,5)
ALT (U/L)	17.9 (12,21)	27.72 (17.33,30.2)
AST (U/L)	23.43 (18.7,26.3)	25.34 (20.5,28.87)
Urea (μmol/L)	4.9 (3.92,5.77)	5.4 (4.45,6.15)
Creatinine (μmol/L)	65.63 (57,73)	88.15 (78.47,94)
eGFR (mL/min/1.73m^2)	114.07 (92.88,129.64)	107.1 (93.98,120.23)
CystatinC (mg/L)	0.95 (0.82,1.06)	0.97 (0.87,1.09)
UA (μmol/L)	301.19 (253.8,337.7)	388.6 (331.95,441.42)
Glucose (mmol/L)	5.55 (4.92,5.83)	5.5 (4.79,5.55)
HbA1C (%)	5.84 (5.5,6)	5.84 (5.5,6.07)
TG (mmol/L)	1.38 (0.89,1.57)	1.79 (1.07,2.16)
TC (mmol/L)	5.36 (4.68,6.07)	5.03 (4.3,5.74)
HDL (mmol/L)	1.46 (1.27,1.61)	1.23 (1.02,1.37)
LDL (mmol/L)	3.41 (2.9,3.9)	3.3 (2.67,3.86)
HDLTCratio	0.28 (0.25,0.31)	0.25 (0.2,0.29)

SBP: systolic blood pressure; DBP: diastolic blood pressure; MAP: mean arterial pressure; HCT: Hematocrit; RBC: red blood cell; Hb: Hemoglobin; MCV: mean corpuscular volume; MCH: mean corpuscular hemoglobin; MCHC: mean corpuscular hemoglobin concentration; RDW: red blood celldistribution width; WBC: white blood cell; Plt: platelet; N: Neutrophil; L: lymphocyte; Eso: eosinophil; Baso: basophile; Mono: monocytes; PCT: procalcitonin; MPV: mean platelet volume; PDW: platelet distribution width; TP: total protein; ALB: albumin; GLOB: globulin; AGratio: albumin-globulin ratio; TBIL: total bilirubin; DBIL: direct bilirubin; TBA: Total biliary acid; ALT: alanine transaminase; AST: aspartate aminotransferase; eGFR: estimated glomerular filtration rate; UA: uric acid; HbA1C: glycated haemoglobin; TG: Triglyceride; TC: total cholesterol; HDL: high density lipoprotein; LDL: low density lipoprotein; HDLTCration: the ratio of high density lipoprotein-globulin to total cholesterol. IQR: Interquartile Range.

Supplementary Table S2. Correlations between different functional changes scores

Variable1	Variable2	Cor	P value
RBCscore	BPcore	-0.2	0.000226908
RBCscore	Renalscore	0.15	0.006322505
BPcore	Renalscore	0.13	0.016322877
Liverscore	Renalscore	0.03	0.551907645
RBCscore	Liverscore	0.02	0.681441015
Liverscore	BPcore	0.05	0.392655568

BPscore: blood pressure change socre; Liversocore: liver functional change score; Renalscore: renal functional change score; RBCscore: RBC functional change score.

Supplementary Table S3. The complete blood count tests of *eBpgm* $^{-/-}$ and *Bpgm* $^{f/f}$ mice.

Variable	4-month-old *Bpgm* $^{f/f}$ mice	4-month-old *eBpgm* $^{-/-}$ mice	15-month-old *Bpgm* $^{f/f}$ mice	15-month-old *eBpgm* $^{-/-}$ mice
WBC	5.13±1.41	3.58±0.62*	3.61±1.21	3.96±1.51
Neu	2.34±1.02	1.63±0.78	1.82±0.98	1.82±0.42
Lym	2.69±0.99	1.3±0.57*	1.65±1.21	2.94±1.60
Mon	0.14±0.07	0.17±0.17	0.08±0.03	0.4±0.28$^{\#}$
Eos	0.16±0.07	0.06±0.03**	0.06±0.05	0.23±0.15$^{\#\#}$
Bas	NA	NA	NA	NA
Neu%	43.8±13.38	49.49±14.8	52.97±22.01	38.15±16.38
Lym%	50.74±13.07	43.59±17.88	43.13±21.27	50.37±17.01
Mon%	2.54±1.22	4.79±4.32	2.25±0.79	7.57±3.89$^{\#}$
Eos%	2.87±0.78	2.08±1.17	1.58±1.01	3.81±1.74$^{\#\#}$
Bas%	0.04±0.05	0.06±0.05	0.07±0.12	0.1±0.15
RBC	7.27±0.58	7.77±0.43*	6.84±0.68	7.52±0.48$^{\#}$
Hb	111.56±8.76	116.38±5.48*	102±9.01	116±8.00$^{\#}$
HCT	36.38±2.75	38.55±1.98*	33.03±2.94	36.88±2.61$^{\#}$
MCV	48.18±1.56	49.9±1.98	48.33±1.18	50.94±1.53$^{\#}$
MCH	14.74±0.55	15.08±0.48	14.9±0.21	15.46±0.57$^{\#}$
MCHC	306.67±3.71	302±4.38	308.83±7.28	303.4±5.70
RDW-CV	16.58±1.33	15.86±0.94	17.48±2.09	16.36±1.22
RDW-SD	28.01±2.57	27.84±2.40	29.58±4.06	29.25±2.45
PLT	768.11±250.13	797.25±164.82	888.17±325.2	927.4±281.42
MPV	5.76±0.27	5.56±0.23	5.5±0.19	5.62±0.21
PDW	15.88±0.27	15.6±0.35	15.57±0.32	15.57±0.30
PCT	0.44±0.14	0.44±0.08	0.49±0.19	0.52±0.15

*4-month-old *Bpgm*$^{f/f}$ vs 4-month-old *eBpgm*$^{-/-}$, $^{\#}$15-month-old *Bpgm*$^{f/f}$ vs 15-month-old *eBpgm*$^{-/-}$. Data are represented as mean ± SD. $^{*}p<0.05$, $^{**}p<0.01$, $^{***}p<0.001$; $^{\#}p<0.05$, $^{\#\#}p<0.01$, $^{\#\#\#}p<0.001$.

Supplementary Table S4. The complete blood count tests of *eENT1*$^{-/-}$ and *ENT1* $^{f/f}$ mice.

Variable	4-month-old *ENT1* $^{f/f}$ mice	4-month-old *eENT1*$^{-/-}$ mice	15-month-old *ENT1* $^{f/f}$ mice	15-month-old *eENT1*$^{-/-}$ mice
WBC	2.64±0.64	2.51±0.75	2.78±0.86	2.8±1.10
Neu	0.98±0.40	0.98±0.48	1.25±0.72	1.36±0.81
Lym	1.42±0.68	1.28±0.31	1.35±0.56	1.18±0.32
Mon	0.12±0.06	0.16±0.15	0.09±0.06	0.18±0.18
Eos	0.12±0.10	0.09±0.05	0.09±0.05	0.09±0.05
Bas	NA	NA	NA	NA
Neu%	38.23±14.00	36.14±17.12	44.94±12.94	45.69±12.52
Lym%	52.33±17.93	54.21±15.71	48.14±11.84	45.08±13.57
Mon%	4.92±3.37	6.19±4.17	3.58±3.54	6.2±4.43
Eos%	4.5±3.11	3.45±1.77	3.34±1.25	3.03±0.90
Bas%	0.02±0.04	0.02±0.04	0.01±0.04	0±0
RBC	8.13±0.59	7.67±0.41$^{\#}$	7.46±0.79	7.20±0.88$^{\#}$
Hb	116.33±10.37	111.45±5.37	110±10.86	106.75±9.98$^{\#}$
HCT	37.38±3.14	36.09±1.51	36.41±3.51	34.33±2.70$^{\#}$
MCV	45.95±0.88	47.08±0.95	48.94±3.13	49.98±2.81$^{\#}$
MCH	14.30±0.37	14.55±0.33	14.78±1.2	14.63±0.63
MCHC	311.17±3.87	308.91±4.25	302.25±11.73	301.75±8.18
RDW-CV	15.32±1.11	16.66±1.22*	17.76±2.35	17.45±1.92
RDW-SD	24.83±1.61	27.64±2.38*	30.63±4.79	29.91±4.65
PLT	1095.33±84.93	997.45±179.44	1076±337.02	1206.42±293.11
MPV	5.2±0.09	5.24±0.20	5.91±0.32	5.75±0.67
PDW	15.07±0.1	15.15±0.22	15.71±0.38	15.51±0.59
PCT	0.57±0.05	0.52±0.09	0.64±0.02	0.68±0.12

*4-month-old *Ent1*$^{f/f}$ vs 4-month-old *eEnt1*$^{-/-}$, $^{\#}$15-month-old *Ent1*$^{f/f}$ vs 15-month-old *eEnt1*$^{-/-}$. Data are represented as mean ± SD. $^{*}p<0.05$, $^{**}p<0.01$, $^{***}p<0.001$; $^{\#}p<0.05$, $^{\#\#}p<0.01$, $^{\#\#\#}p<0.001$.

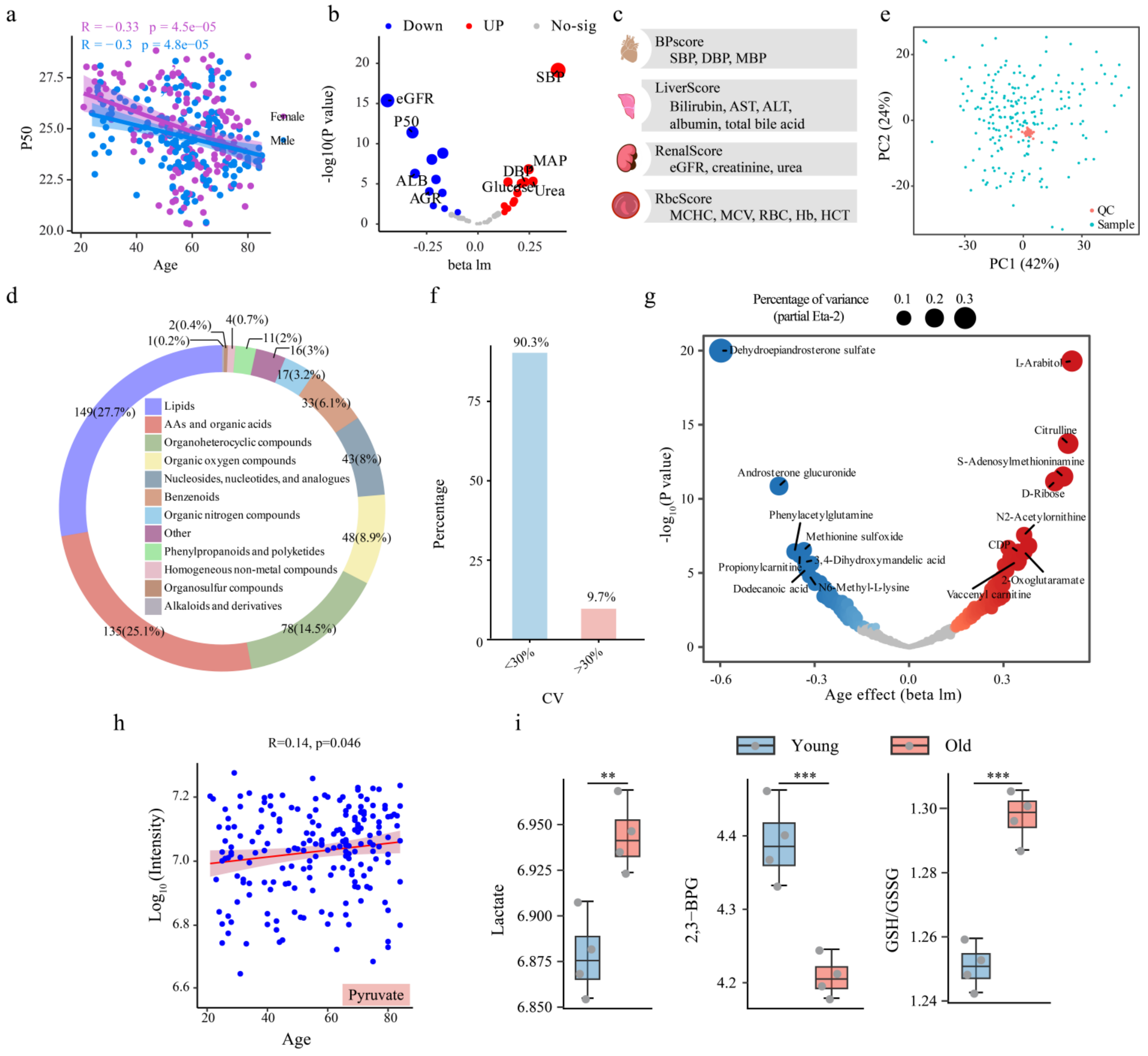

Supplementary Fig S1. Quality assessment of erythrocyte metabolomics profiling and aging-related erythrocyte metabolic characteristics in humans.

(a) Gender differences of P50.

(b) Volcano plot representing changes in clinical parameters with age. The linear analysis, adjusted for BMI and sex, was evaluated using the F-test).

(c) The related clinical parameters of clinical function variation analyses (These parameters are significantly related to age). RBC: red blood cell; Hb: Hemoglobin; MCV: mean corpuscular volume; HCT: Hematocrit; MCHC: mean corpuscular hemoglobin concentration; ALT: alanine transaminase; AST: aspartate aminotransferase; eGFR: estimated glomerular filtration rate.

(d) The PCA score of QC and RBC samples.

(e) Chemical composition of RBC metabolome using the super class from HMDB database.

(f) Variation coefficient distribution of metabolites in QC samples.

(g) Aging-related erythrocyte metabolic characteristics.

(h) The pyruvate change in erythrocytes during aging.

(i) The levels of lactate, 2,3-BPG and the ratio of GSH to GSSG between young and old mice. The young group represents the 4-month-old mice; The old group represents 15-month-old WT mice; N=4. Data are presented as median and quartile

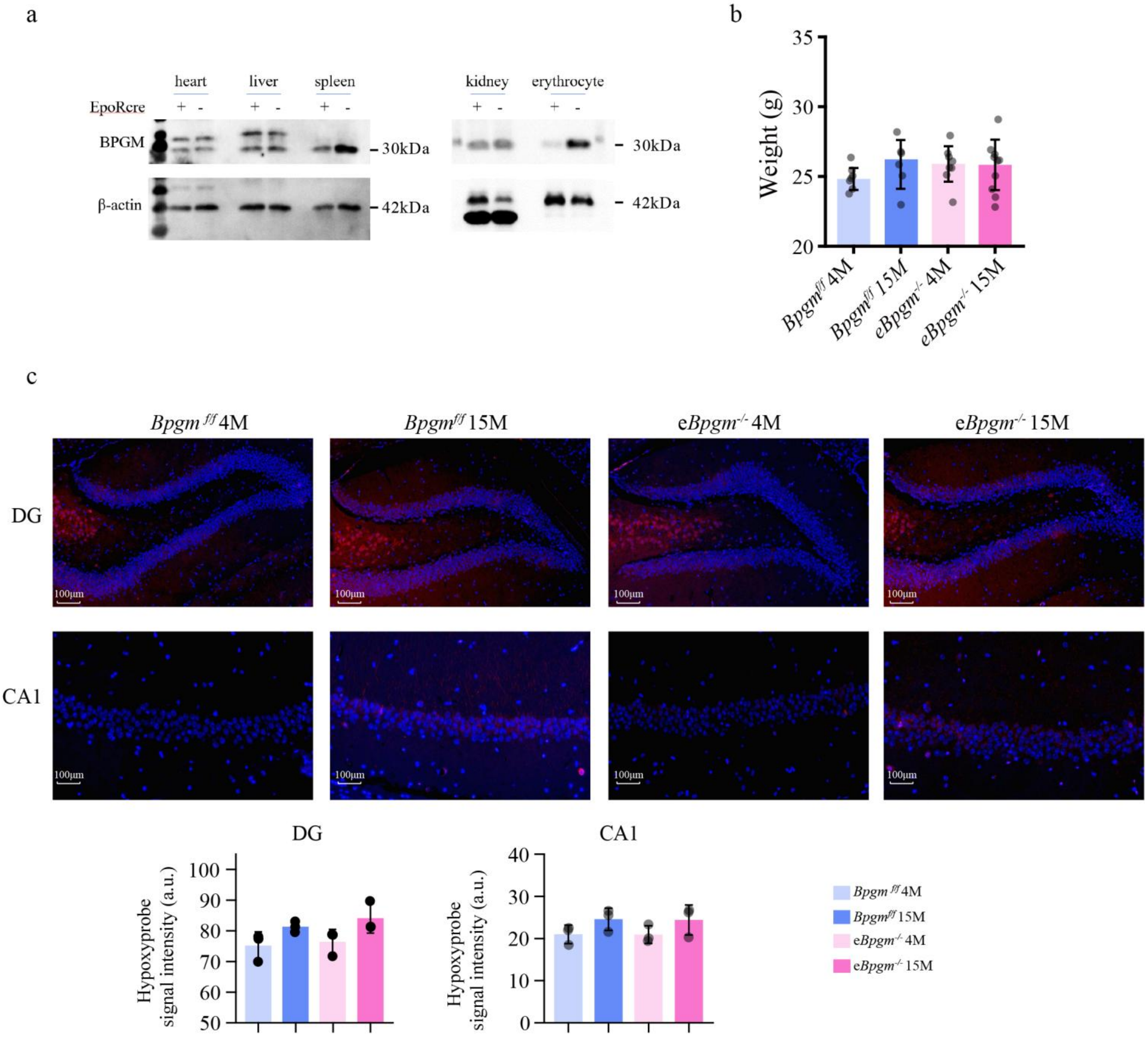

Supplementary Fig S2. Identification of BPGM knockout in erythrocyte by western blot experiment and its aging-related phenotype

(a) Expression level of BPGM protein in erythrocytes and different tissues in both *eBpgm* $^{-/-}$ and *Bpgm* $^{f/f}$.

(b) Body weight changes in *eBpgm* $^{-/-}$ and *Bpgm* $^{f/f}$ mice from young to old.

(c) Hypoxyprobe staining of hippocampus dentate gyrus (DG) and hippocampus CA1, and quantification analysis of hypoxia intensity. The Red color reflects the level of oxygen deficiency; the more intense the redder the color, the greater the oxygen insufficiency. N=3. Data are represented as mean ± SD.

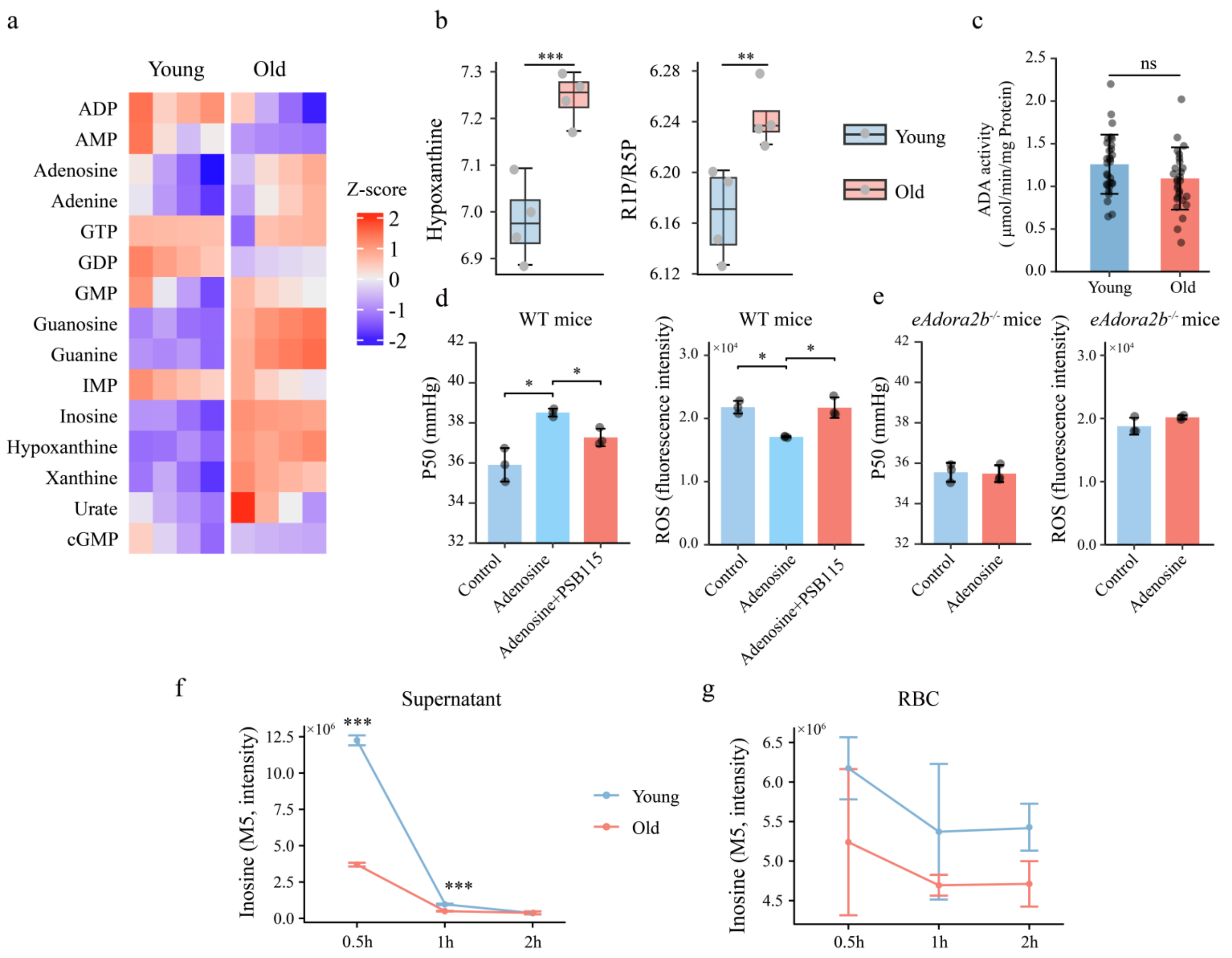

Supplementary Fig S3. The connection between erythrocyte purine metabolism and aging.

(a) The heatmap of purine metabolic intermediates between young and old groups. The young group represents the 4-month-old mice; Old group represents 15-month-old WT mice; n=4

(b) Changes of hypoxanthine and R1P/R5P between young and old mice. N=4. Data are presented as median and quartile

(c) The ADA enzyme activity of RBCs during aging between young (20yrs-30yrs) and old individuals (60yrs-80yrs). Data are represented as mean ± SD.

(d) Changes in P50 and ROS following incubation of 4-month-old WT mouse RBCs with 100 μM adenosine for one hour in the absence or presence of PSB1115 (an ADORA2B inhibitor). N=3, Data are represented as mean ± SD. *p<0.05.

(e) Changes in P50 and ROS following incubation of *eAdora2b*$^{-/-}$ (erythrocyte-specific A2B deficient mice) RBC with 100 μM adenosine. N=3, Data are represented as mean ± SD.

(f, g) The peak intensity of inosine in supernatant (f) and RBCs (g) after incubation of red blood cells with 0.1mM [$^{13}C_5$] inosine for 0.5, 1, and 2h; N = 3 in each group. Data are represented as mean ± SD. *p<0.05, **p<0.01, ***p<0.001.

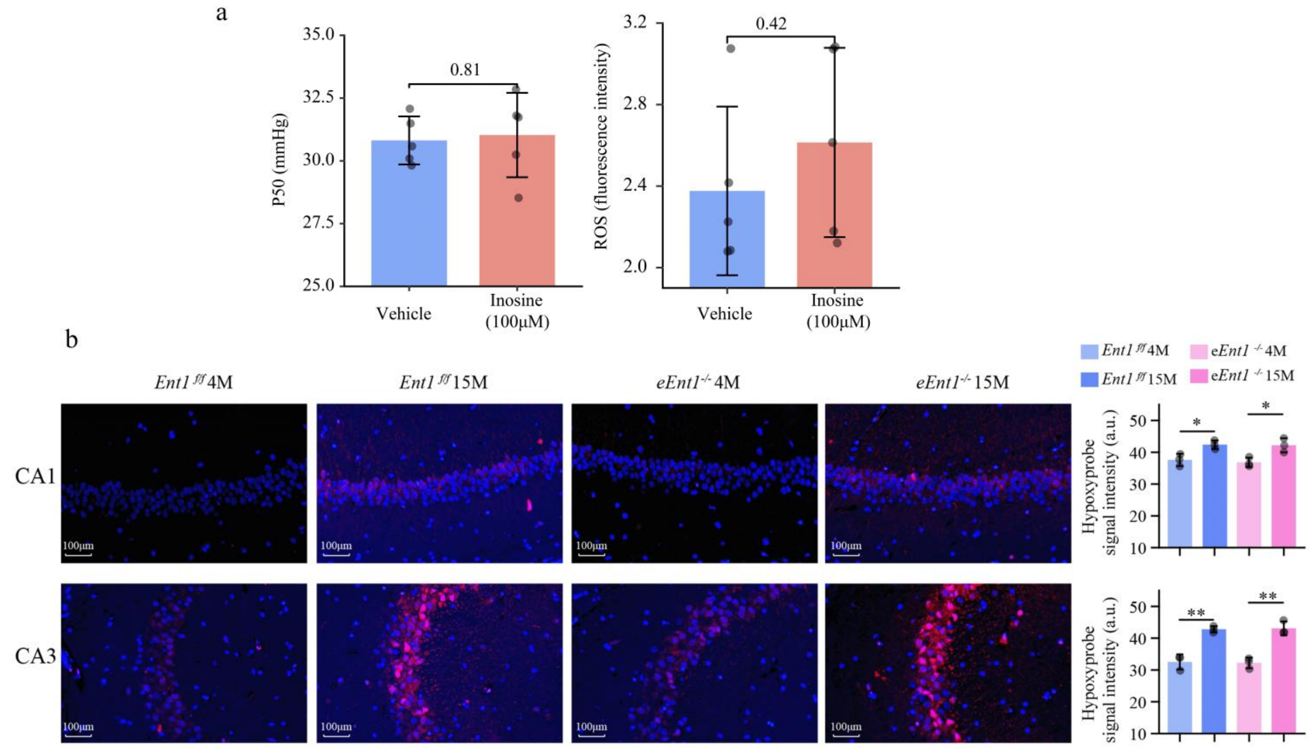

Supplementary Fig S4. Inosine treatment in primary RBCs from *eEnt1*$^{-/-}$ mice and hypoxia levels in the hippocampus of *eEnt1*$^{-/-}$ mice. n=4

(a) Changes in P50 and ROS following treatment of 4-month-old *eENT1*$^{-/-}$ RBCs for 1 hour with 100 μM inosine (n=5).

(b) The hypoxyprobe staining of hippocampus CA1 and CA3, and quantification analysis of hypoxia intensity. Red color reflects the level of oxygen deficiency; the more intense the redder the color, the greater the oxygen deficiency. N = 3 in each group, Data are represented as mean ± SD. *p<0.05, **p<0.01.

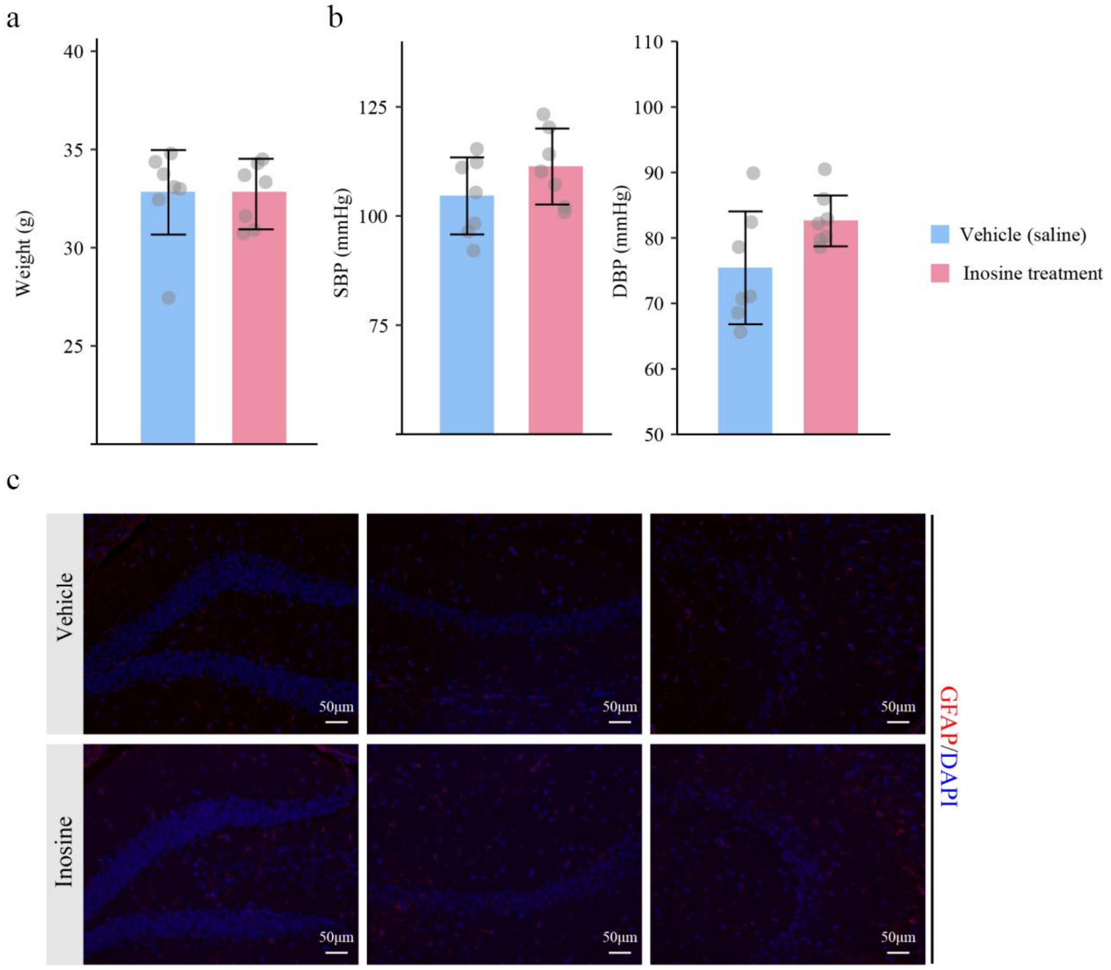

Supplementary Fig S5. The impact of supplemental inosine on weight, blood pressure and astrocytes in the brain.

(a, b) The impact of supplemental inosine on weight (a) and blood pressure (b).

(c) The immunofluorescence staining of GFAPs in different regions (From left to right: DG, CA1, CA3)

Data represented as mean ± SD. N = 7 in each group. SBP: systolic blood pressure; DBP: diastolic blood pressure;

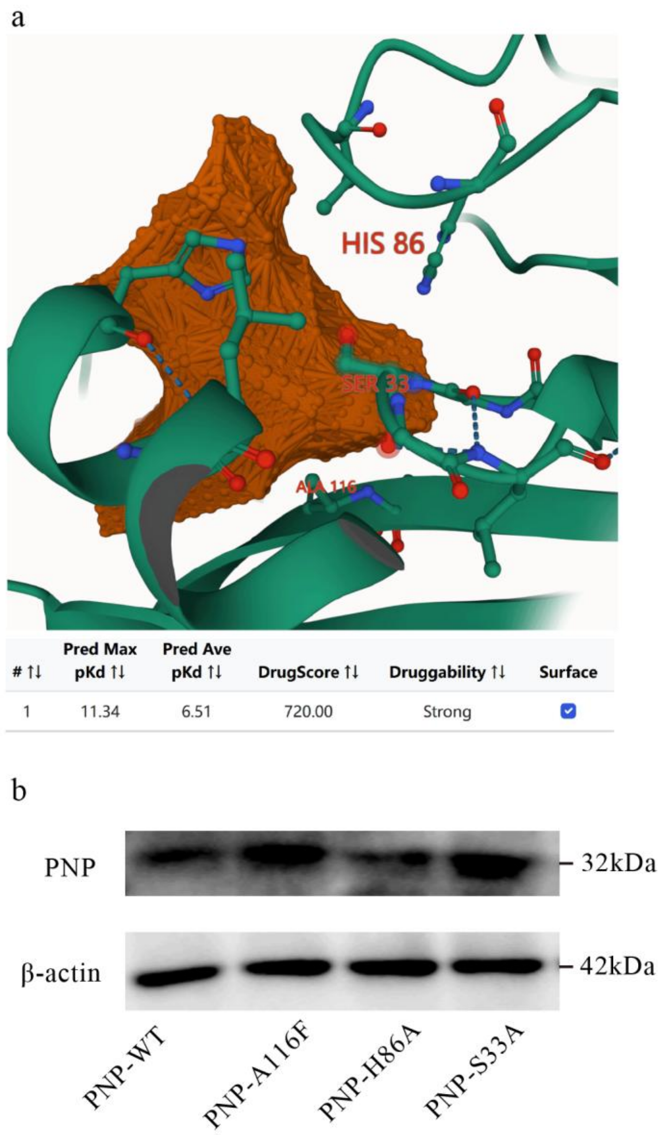

Supplementary Fig S6. Prediction of active pockets of PNP (a) and western blot experiments of 293T cells transfected with PNP plasmid (b). The CavityPlus online tool (https://www.pkumdl.cn/cavityplus) was used for predicting the active pockets of PNP proteins, determining the best active pocket according to the ranking of DrugScore and Druggability.

PNP-WT: Control, A116F, Ala116 mutated to phenylalanine; S33A: Ser33 mutated to alanine; H86A, His86 mutated to alanine.
